# TIGAR deficiency enhances cardiac resilience through epigenetic programming of Parkin expression

**DOI:** 10.1172/jci.insight.200105

**Published:** 2026-02-26

**Authors:** Yan Tang, Stanislovas S. Jankauskas, Li Liu, Xujun Wang, Alus M. Xiaoli, Fajun Yang, Gaetano Santulli, Daorong Feng, Jeffrey E. Pessin

**Affiliations:** 1 Department of Medicine, Norman Fleischer Institute for Diabetes and Metabolism,; 2 Department of Developmental Biology, and; 3 Department of Molecular Pharmacology, Albert Einstein College of Medicine, Bronx, New York, USA.

**Keywords:** Cardiology, Cell biology, Metabolism, Diabetes, Heart failure, Mitochondria

## Abstract

Mitochondrial dysfunction devastates the heart in major cardiovascular diseases, yet the mechanisms governing mitochondrial quality control remain elusive. We discovered that TIGAR (TP53-induced glycolysis and apoptosis regulator) deficiency established profound cardiac protection through developmental epigenetic programming of Parkin expression. Using mice with whole-body and cardiomyocyte-specific TIGAR knockout, we demonstrated remarkable cardioprotection following myocardial infarction with maintained ejection fraction, and complete resistance to diet-induced cardiac hypertrophy despite comparable weight gain. TIGAR deficiency triggered dramatic increases in Parkin expression across all somatic tissues except testes, where Parkin levels remained extraordinarily high (100-fold greater than cardiac levels) regardless of TIGAR status, revealing tissue-specific regulatory mechanisms. This protection was entirely Parkin dependent, as double-knockout mice lost all cardioprotective benefits. Crucially, adult TIGAR manipulation failed to alter Parkin levels, demonstrating that this pathway operated exclusively during critical developmental windows to program lifelong cardiac resilience. Whole-genome bisulfite sequencing identified reduced DNA methylation in *Prkn* intron 10 as the key regulatory mechanism, with CRISPR deletion dramatically increasing Parkin expression in multiple cell lines. Our findings reveal how early cardiac metabolism programs lifelong cardiac function through epigenetic mechanisms, and identify developmental metabolic programming as a potential therapeutic target for preventing both ischemic heart disease and metabolic cardiomyopathy.

## Abstract 2

This study has uncovered a novel regulatory pathway occurs during cardiac development that can be manipulated to provide lifelong cardioprotection via enhanced mitochondrial quality control.

## Introduction

Cardiovascular disease remains the leading cause of death worldwide, with mitochondrial dysfunction at the center of major cardiac pathologies (1–4). The heart, as one of the most metabolically active organs, requires exceptional mitochondrial function to meet its enormous energy demands, consuming approximately 6 kg of ATP daily (5). Impaired mitochondrial quality control mechanisms have serious consequences for cardiac performance and stress survival.

In ischemic heart disease, mitochondrial dysfunction drives cardiac pathology through interconnected mechanisms. During myocardial infarction (MI), mitochondrial damage leads to accumulation of dysfunctional organelles, increased reactive oxygen species production, and release of mitochondrial damage-associated molecular patterns that trigger robust inflammatory responses (6, 7). These deleterious processes create a vicious cycle in which mitochondrial damage propagates cellular dysfunction beyond the original injury into viable myocardium, contributing to adverse remodeling and eventual heart failure.

The complexity is equally evident in diabetic heart disease, where mitochondrial dysfunction precedes overt cardiac functional abnormalities (4, 8, 9). Even with optimal glycemic control, diabetic patients remain at increased risk of heart failure due to fundamental alterations in cardiac energy metabolism, including preferential utilization of fatty acid over glucose, impaired energetic efficiency, and progressive mitochondrial dysfunction. This metabolic inflexibility renders diabetic hearts particularly vulnerable to additional stressors and limits adaptive capacity during increased demands.

Mitochondrial quality control mechanisms, particularly selective autophagy of damaged mitochondria (mitophagy), represent critical cardioprotective pathways (4). The PINK1/Parkin pathway constitutes the primary mechanism governing mitophagy in mammalian cells (10). Under normal conditions, PTEN-induced kinase 1 (PINK1) is imported into healthy mitochondria and rapidly degraded. However, when mitochondrial membrane potential is compromised, PINK1 accumulates on the outer mitochondrial membrane, where it phosphorylates ubiquitin and recruits the E3 ubiquitin ligase Parkin (11). Activated Parkin then ubiquitinates numerous outer mitochondrial membrane proteins, marking damaged organelles for selective autophagic degradation.

The cardiac significance of this pathway has been demonstrated across multiple experimental models and disease states. Parkin-deficient mice exhibit exacerbated cardiac dysfunction following MI, with increased infarct size, impaired functional recovery, and accelerated progression to heart failure (12, 13). Conversely, cardiac-specific Parkin overexpression or pharmacological enhancement of Parkin activity provides substantial cardioprotection, preserving cardiac function and improving survival following ischemic injury (14–16).

Postnatal cardiac development involves dramatic metabolic reprogramming from glycolysis-dependent fetal metabolism to fatty acid oxidation–dependent adult metabolism. This transition requires extensive mitochondrial biogenesis, maturation of respiratory complexes, and establishment of robust quality control mechanisms. Parkin plays a crucial role in this developmental process, with Parkin-mediated mitophagy directing perinatal cardiac metabolic maturation (17). Disruption of this developmental programming through genetic deletion of key mitochondrial regulators including Parkin, TFAM, or PGC-1α results in severe cardiac dysfunction and often perinatal lethality (18, 19).

The concept of developmental programming suggests that early environmental influences can establish persistent phenotypic changes that affect disease susceptibility throughout life (20, 21). In the cardiovascular system, evidence indicates that metabolic conditions during critical developmental windows can permanently alter cardiac structure, function, and stress resistance (22, 23). However, the molecular mechanisms underlying such programming, particularly those involving mitochondrial quality control, remain incompletely understood.

TP53-induced glycolysis and apoptosis regulator (TIGAR) functions as a multifaceted regulator of cellular metabolism and stress responses. Originally identified as a p53 target gene (24), TIGAR exhibits both phosphatase activity toward fructose-2,6-bisphosphate and additional regulatory functions influencing cellular energetics, redox homeostasis, and stress resistance (25). In cardiac pathophysiology, TIGAR’s role appears to be context dependent. The p53/TIGAR axis can exacerbate cardiac damage after MI (26), while TIGAR ablation preserves myocardial energetics in pressure overload heart failure and reduces pathological cardiac hypertrophy (27, 28).

Our previous investigations revealed that TIGAR deficiency enhances TCA cycle flux, increases mitochondrial respiratory capacity, and exhibits antiinflammatory properties (29, 30). These metabolic effects suggested potential connections to mitochondrial quality control mechanisms, prompting us to investigate relationships between TIGAR and Parkin-mediated mitophagy in cardiac protection.

In this study, we demonstrate that TIGAR-knockout mice exhibit dramatically increased Parkin expression through developmental epigenetic programming, conferring significant cardioprotection against both acute ischemic injury and chronic metabolic stress. Using Parkin/TIGAR double-knockout models, we confirmed that these protective effects depend on Parkin. Whole-genome bisulfite sequencing revealed specific differentially methylated regions in the Parkin gene body that regulate its expression.

## Results

### TIGAR deficiency confers comprehensive cardioprotection against MI.

**Figure 1:**
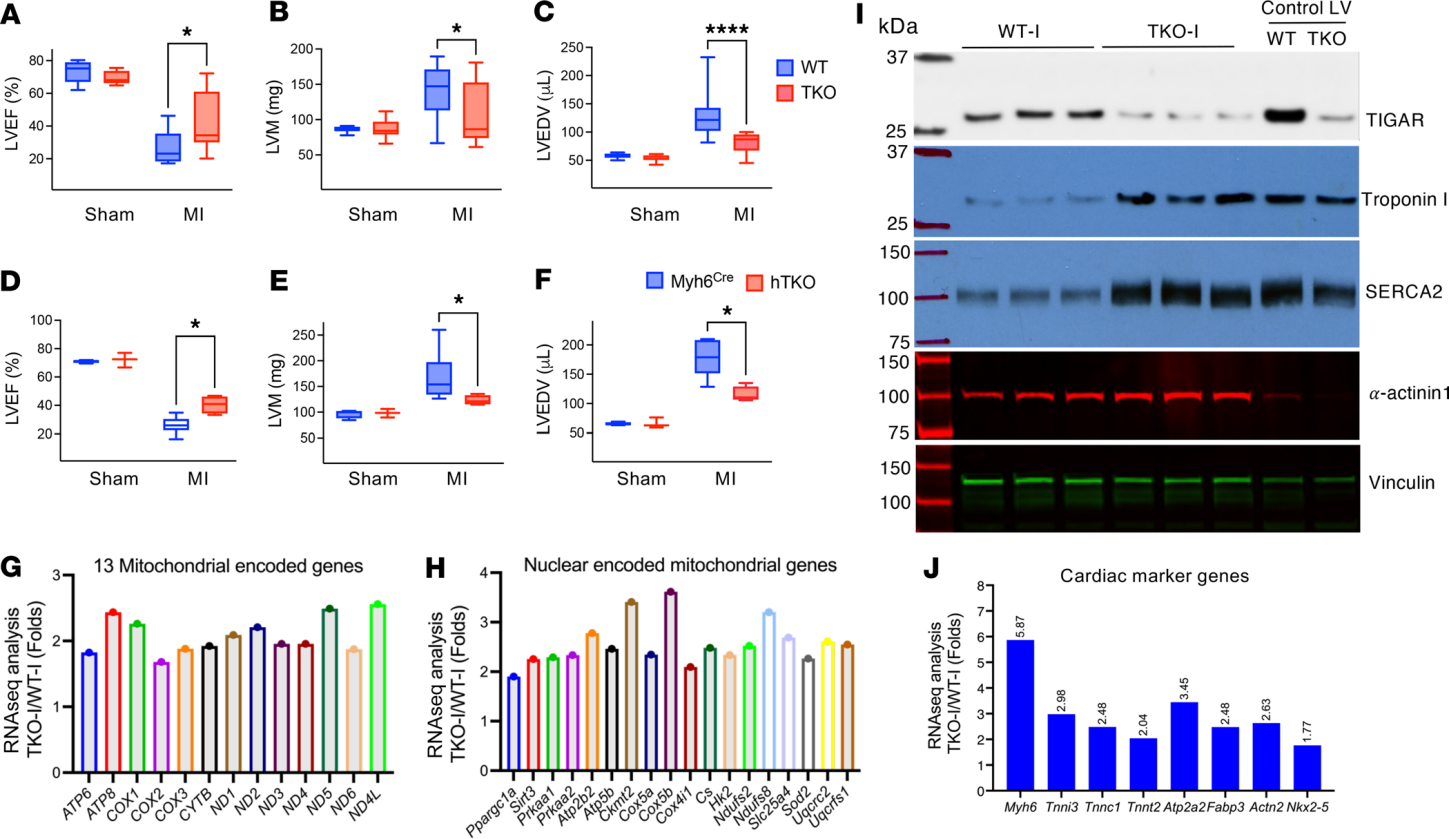
TIGAR knockout protects against post–myocardial infarction cardiac dysfunction. (**A**–**C**) Echocardiograms and quantification of left ventricular ejection fraction (LVEF), mass (LVM), and end-diastolic volume (LVEDV) in 4-month-old male WT (*n* = 10) and TKO mice (*n* = 13) 4 weeks after myocardial infarction (MI). MI was induced by left anterior descending (LAD) artery ligation. Sham-operated mice served as controls. (**D**–**F**) Echocardiograms and quantification of left ventricular ejection fraction (LVEF), mass (LVM), and end-diastolic volume (LVEDV) in 4-month-old male control Myh6^Cre^ mice (*n* = 6) and hTKO mice (*n* = 4) 4 weeks after MI. (**G** and **H**) RNA-seq analysis of mitochondrial-encoded genes (**G**) and nuclear-encoded mitochondrial genes (**H**) from the infarct zone, expressed as fold changes in TKO versus WT. (**I**) Immunoblot analysis of TIGAR, troponin I, SERCA2, α-actinin-1, and vinculin in infarcted heart tissue from WT and TKO mice, with normal left ventricular tissue (Control LV) as reference. (**J**) RNA-seq analysis of cardiac marker genes from the infarct zone, expressed as fold changes in TKO versus WT. For RNA-seq analyses (**G**, **H**, and **J**), RNA from 3 mice per group was pooled for sequencing. Data represent mean ± SD. Statistical significance was determined by 1-way ANOVA. *P < 0.05; ****P < 0.0001.

TIGAR-deficient mice demonstrated comprehensive cardioprotection against ischemic injury. Analysis of both mice with whole-body TIGAR knockout (TKO) and mice with heart cardiomyocyte–specific TIGAR knockout (hTKO) distinguished between cardiomyocyte-intrinsic effects and systemic metabolic changes (Figure 1 and Supplemental Figure 1; supplemental material available online with this article; https://doi.org/10.1172/jci.insight.200105DS1). Following permanent left anterior descending coronary artery occlusion, both knockout models showed remarkably preserved cardiac function at 4 weeks after injury. Our echocardiographic analysis revealed substantial preservation of cardiac function. TKO mice maintained ejection fraction at 43.35% ± 17.76% (*n* = 13), while WT controls exhibited severely impaired function at 26.36% ± 9.83% (*n* = 10, *P* < 0.05) (Figure 1A). This level of protection was substantial, representing retention of approximately 60% of baseline cardiac function compared with only 36% in controls. Cardiomyocyte-specific knockout (hTKO) mice demonstrated similar protection with ejection fraction at 40.46% ± 6.29% compared with control mice at 26.06% ± 6.08% (Figure 1D). These findings indicated that cardioprotection resulted primarily from cardiomyocyte-intrinsic mechanisms rather than systemic metabolic alterations.

Both knockout models were significantly protected against increases in left ventricular mass and volume that typically follow MI — key indicators of harmful cardiac remodeling (Figure 1, B, C, E, and F). WT mice showed typical post-infarction cardiac remodeling, while knockout animals maintained relatively normal overall cardiac structure despite similar-sized infarct areas. This preservation was accompanied by significantly reduced cardiac enlargement in TKO hearts after MI compared with WT hearts (Supplemental Figure 2, A and B). After MI, fibrotic processes typically extend into non-infarcted areas and alter cardiac structure (31, 32). However, post-infarction fibrotic processes were notably restricted in non-infarct regions of TKO hearts, coinciding with reduced expression of periostin and galectin-3 (Supplemental Figure 3, A and B).

Our RNA sequencing analysis of infarct territories revealed extensive preservation of mitochondrial gene expression in TKO hearts compared with WT controls (Figure 1G). Notably, all 13 mitochondrial DNA–encoded genes showed significant preservation in TKO hearts, contrasting sharply with the widespread suppression observed in WT infarct tissue (7, 33). This pattern extended to nuclear genes affecting mitochondrial function, with maintained expression of genes involved in respiratory complexes and PGC-1α, a master regulator of mitochondrial biogenesis (Figure 1H). In contrast, there was no significant change in the mitochondrial DNA–encoded gene expression in either the border or remote zones between the WT and TKO infarcted hearts (Supplemental Figure 3, C and D). Similarly, there was no consistent change in expression of the nuclear-encoded genes in the border or remote zones with the notable exception of *Atp2b2* mRNA (Supplemental Figure 3, E and F). Pathway analyses indicated highly significant differences in mitochondria functional pathway preservation in the infarct zone between WT and TKO mice that were generally distinct from the border and remote zones (Supplemental Figure 4, A–C). The preservation of mitochondrial gene expression and pathways paralleled findings from pharmacological studies using the Parkin activator PR-364 (16), suggesting a common mechanism involving enhanced mitochondrial quality control. Western blot analysis demonstrated remarkable preservation of cardiomyocyte-specific proteins within TKO infarct zones. Cardiac troponin I (cTnI), a highly specific cardiac marker, was markedly reduced in WT infarct zones but maintained at near-normal levels in TKO infarct areas. Similarly, SERCA2a protein, crucial for cardiac contractility through calcium handling regulation, showed dramatic preservation in TKO hearts (Figure 1I and Supplemental Figure 5).

The preservation of both structural and functional proteins suggested enhanced cardiomyocyte survival within infarct territories. RNA sequencing quantification confirmed partially maintained expression of key cardiac genes including *Myh6* (α-myosin heavy chain), *Tnni3* (cardiac troponin I), *Tnnc1* (cardiac troponin C), *Tnnt2* (cardiac troponin T), *Actn2* (α-actinin-2), *Nkx2-5*, and *Atp2a2* (SERCA2a) in TKO infarct zones (Figure 1J). The preservation of these diverse cardiac markers indicated protection against multiple forms of cardiomyocyte death, including both apoptosis and necrosis. RNA-seq quantification also confirmed that ketolysis-related genes and fatty acid oxidation gene clusters were partially preserved in TKO infarct zones (Supplemental Figure 6, A and B).

### Parkin upregulation as the central mediator of cardioprotection.

**Figure 2:**
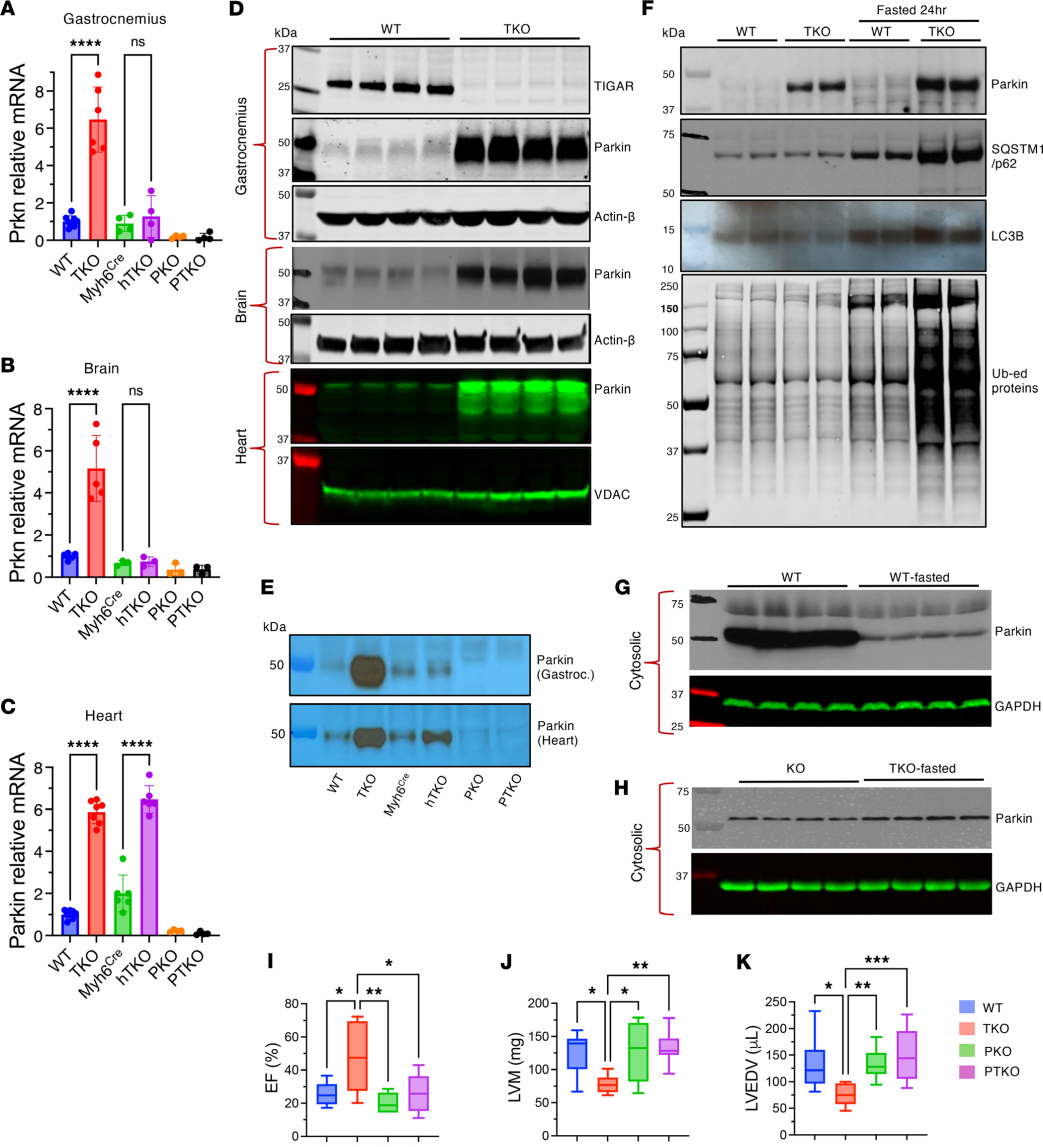
Parkin upregulation mediates cardiac protection in TKO and hTKO mice. (**A**–**C**) Parkin (*Prkn*) mRNA levels in gastrocnemius muscle (**A**), brain (**B**), and heart (**C**) of WT, TKO, Myh6^Cre^, hTKO, Parkin-knockout (PKO), and Parkin/TIGAR double-knockout (PTKO) mice determined by RT-qPCR. Data normalized to WT expression. (**D**) Western blot analysis of TIGAR and Parkin protein levels in gastrocnemius muscle, brain, and heart tissues from WT and TKO mice, with β-actin and VDAC as loading controls. (**E**) Detection of Parkin protein in gastrocnemius muscle and heart tissues from WT, TKO, Myh6^Cre^, hTKO, PKO, and PTKO mice by immunoprecipitation followed by immunoblotting. (**F**) Western blot analysis of mitophagy-related proteins, including Parkin, SQSTM1/p62, LC3B, and ubiquitinated proteins in heart mitochondrial fractions from fed and 24-hour-fasted WT and TKO mice. (**G** and **H**) Immunoblot analysis of Parkin protein levels in cytosolic fractions from heart tissues of fed and 24-hour-fasted WT (**G**) and TKO (**H**) mice, with GAPDH as loading control. (**I**–**K**) Echocardiographic assessment of left ventricular ejection fraction (EF) (**I**), mass (LVM) (**J**), and end-diastolic volume (LVEDV) (**K**) in 4-month-old male WT, TKO, PKO, and PTKO mice 4 weeks after MI. Data represent mean ± SD. Statistical significance was determined by 1-way ANOVA. *P < 0.05; **P < 0.01; ***P < 0.005; ****P < 0.0001; *n* = 5–10 per group.

Our investigation of molecular mechanisms underlying cardiac protection revealed dramatic Parkin upregulation across multiple tissues in TKO mice, providing insight into the mechanistic basis of observed cardioprotection. Quantitative reverse transcriptase PCR (RT-qPCR) analysis demonstrated significant increases in Parkin (*Prkn*) mRNA expression across multiple tissues. Both gastrocnemius muscle and heart tissue exhibited 6-fold increases (*n* = 7–8, *P* < 0.0001), while brain tissue showed 5-fold elevation (*n* = 5, *P* < 0.001), compared with WT controls (Figure 2, A–C). The magnitude and consistency of these changes across diverse tissue types suggested fundamental alteration in Parkin gene regulation rather than tissue-specific adaptive responses. Importantly, cardiomyocyte-specific TIGAR-knockout (hTKO) mice also demonstrated significant Parkin upregulation in heart tissue (3-fold increase, *n* = 6, *P* < 0.0001) without corresponding changes in non-cardiac tissues such as gastrocnemius muscle or brain. This tissue-specific pattern in hTKO mice confirmed that TIGAR’s regulation of Parkin expression was cell autonomous and did not require systemic TIGAR deficiency.

Western blot analyses confirmed these findings at the protein level, showing substantially elevated Parkin protein expression in TKO mice across all examined tissues (Figure 2D). Because of dramatic differences in Parkin protein levels between WT and TKO samples, immunoprecipitation studies were necessary to concentrate Parkin protein from WT samples for accurate quantitative comparisons (Figure 2E). These studies confirmed substantial upregulation in both TKO and hTKO hearts and skeletal muscle, but not in skeletal muscle from hTKO mice, consistent with mRNA expression patterns.

The functional significance of Parkin upregulation became evident during metabolic stress conditions. Under baseline fed conditions, Parkin phosphorylation at serine 65 (a marker of Parkin activation) was proportionally increased in TKO hearts relative to total Parkin levels, indicating functional competence of upregulated Parkin (Supplemental Figure 7A). During 24-hour fasting, TKO hearts exhibited enhanced mitophagic responses compared with WT controls, evidenced by increased mitochondrial protein ubiquitination, elevated recruitment of autophagy adaptor proteins (SQSTM1/p62), and enhanced LC3B lipidation (Figure 2F). These enhanced responses occurred without baseline depletion of mitochondrial proteins, indicating appropriate regulation without excessive mitochondrial loss (Supplemental Figure 6B).

In the basal state, Parkin is primarily localized to the cytoplasm and translocates to the mitochondria when mitophagy becomes activated (34). In WT hearts, Parkin was depleted from the cytosolic fraction after 24 hours of fasting (Figure 2G), while TKO hearts maintained cytosolic Parkin reserves (Figure 2H). This resulted in greater mitochondria-associated Parkin in both basal and fasted states in TKO hearts (Figure 2F). Despite increased Parkin protein levels, Parkin remains inactive until an appropriate cardiac mitochondrial stress is induced, then provides enhanced capacity for mitochondrial ubiquitination and mitophagy completion.

To definitively establish Parkin’s role in mediating cardioprotective effects, Parkin/TIGAR double-knockout (PTKO) mice were generated. These animals showed complete loss of the protective phenotype previously observed in TKO mice, providing compelling genetic evidence for Parkin’s essential role. Following MI, PTKO mice exhibited severely impaired cardiac function, with post-infarction ejection fraction (25.77% ± 11.61%, *n* = 10) comparable to that in WT mice (25.32% ± 7.25%, *n* = 5) and significantly worse than that in TKO mice (46.94% ± 20.47%, *n* = 9, *P* = 0.012) (Figure 2I). Similarly, parameters of left ventricular remodeling in PTKO mice closely resembled those in WT animals; left ventricular mass and end-diastolic volume were significantly increased compared with those in TKO mice but indistinguishable from those in WT controls (Figure 2, J and K). These findings provided definitive genetic proof that Parkin upregulation was both necessary and sufficient for cardioprotective effects conferred by TIGAR deficiency. The complete reversal of protection in PTKO mice eliminated the possibility that alternative pathways could compensate for Parkin’s absence in the setting of TIGAR deficiency.

### Comprehensive protection against diet-induced metabolic cardiomyopathy.

**Figure 3:**
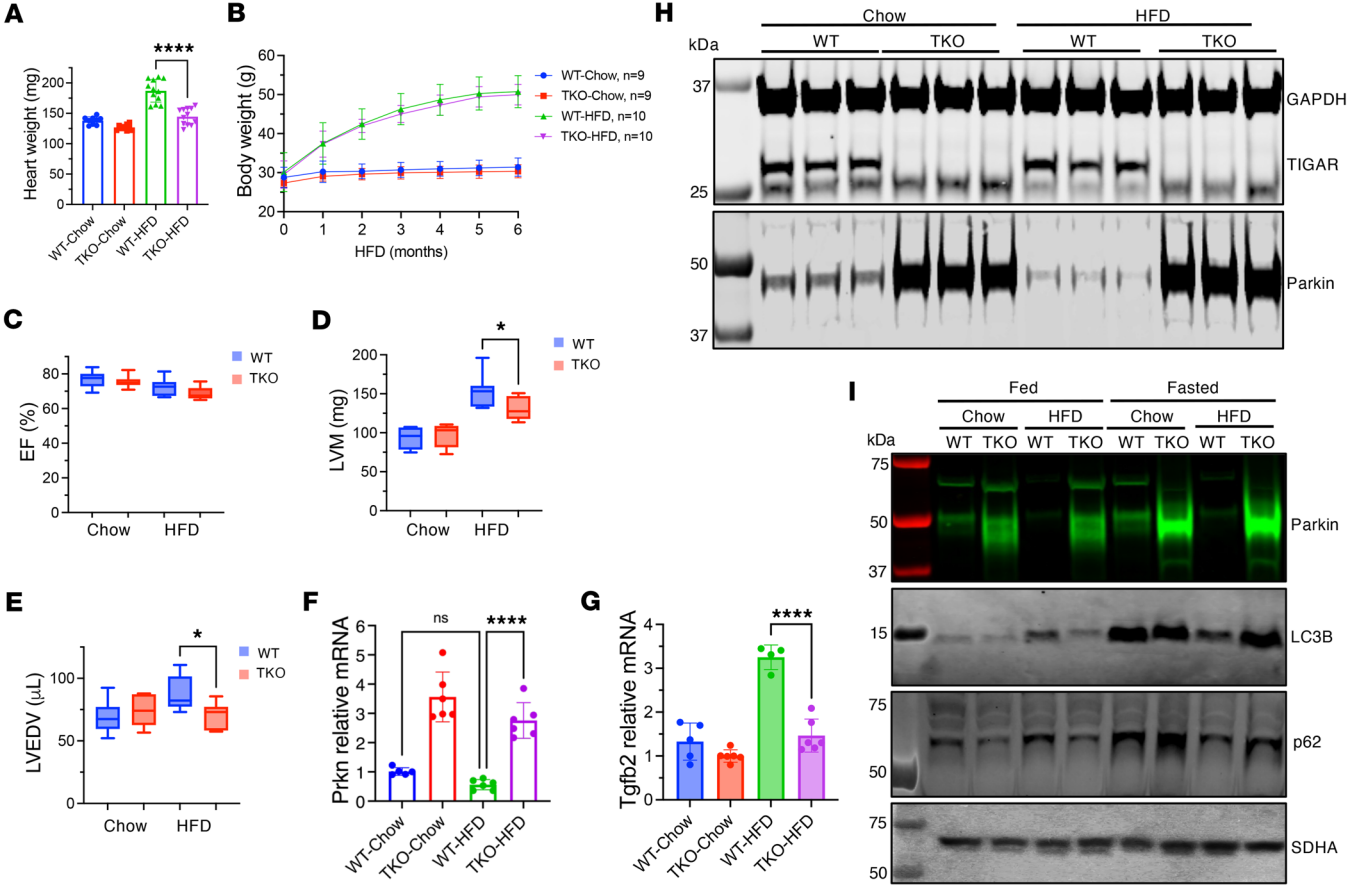
High-fat diet effects on cardiac function and mitophagy in WT and TKO mice. (**A**) Heart weights of WT and TKO mice after 6 months of normal chow or high-fat diet (HFD). HFD increased cardiac mass in WT but not TKO mice. (**B**) Body weight progression in WT and TKO mice during 6 months of normal chow or HFD (*n* = 9–10 per group). (**C**–**E**) Echocardiographic assessment of left ventricular ejection fraction (EF) (**C**), mass (LVM) (**D**), and end-diastolic volume (LVEDV) (**E**) in WT and TKO mice fed normal chow or HFD. (**F** and **G**) mRNA expression levels of Parkin (**F**) and TGF-β2 (**G**) in heart tissue from WT and TKO mice fed normal chow or HFD. Data normalized to WT-chow expression. *n* = 4–6. (**H**) Western blot analysis of TIGAR and Parkin protein levels in heart tissue cytosolic fraction from WT and TKO mice fed normal chow or HFD, with GAPDH as loading control. (**I**) Western blot analysis of mitophagy-related proteins (Parkin, LC3B, and p62) in mitochondrial fractions of heart tissue from fed and 24-hour-fasted WT and TKO mice maintained on normal chow or HFD. SDHA serves as mitochondrial fraction loading control. Data represent mean ± SD. Statistical significance was determined by 1-way ANOVA. *P < 0.05; ****P < 0.0001.

TIGAR has a complex role in metabolic regulation, with studies showing that TIGAR suppresses glycolysis by degrading fructose-2,6-bisphosphate (24), increases glycolysis with reduction of mitochondrial reactive oxygen species by stimulating hexokinase 2 (35), and suppresses inflammatory signaling by inhibiting the linear ubiquitination complex LUBAC (29). Consistent with TIGAR suppressing inflammatory signaling, the TKO mice displayed impaired glucose tolerance (Supplemental Figure 7C). To determine whether the cardioprotective effects of TIGAR deficiency extended beyond acute ischemic injury to chronic metabolic stress conditions, mice were subjected to a 6-month high-fat diet regimen containing 60% calories from fat to model diet-induced metabolic cardiomyopathy. WT mice developed a significantly increased heart weight (182.7 ± 17.2 mg, *n* = 10) compared with chow-fed controls (137.9 ± 6.4 mg, *n* = 9). In striking contrast, TKO mice remained remarkably resistant with only a minimal cardiac mass increase (142 ± 13.5 mg, *n* = 10) compared with chow-fed TKO counterparts (126.8 ± 6.4 mg, *n* = 9) (Figure 3A).

Importantly, this protection occurred despite comparable body weight gains between genotypes (WT high-fat diet, 50.3 ± 5.0 g, vs. TKO high-fat diet, 50.2 ± 1.7 g; *n* = 10), indicating that cardioprotective effects were independent of systemic metabolic load or differences in diet-induced obesity (Figure 3B).

Our echocardiographic analysis revealed that systolic function parameters remained preserved across all groups (Figure 3C), consistent with heart failure with preserved ejection fraction (36). WT mice on high-fat diet exhibited pathological hypertrophic remodeling, including increased left ventricular mass (152.5 ± 20.1 mg vs. 131.0 ± 14.2 mg in TKO, *P* < 0.05) and elevated end-diastolic volume (87.5 ± 13.67 μL vs. 69.89 ± 10.03 μL in TKO, *P* < 0.05) (Figure 3, D and E). TKO hearts maintained normal cardiac geometry, suggesting protection against pathological remodeling. Molecular analysis revealed distinct transcriptional and protein expression profiles corresponding to observed phenotypic differences. WT mice subjected to high-fat diet showed a downward trend in cardiac Parkin mRNA expression (Figure 3F), while Parkin protein levels were significantly decreased (Figure 3H), consistent with previous reports of impaired mitochondrial quality control in metabolic cardiomyopathy.

This Parkin suppression was accompanied by upregulation of established pathological remodeling markers, including transforming growth factor-β1 (*Tgfb1*), a key mediator of cardiac fibrosis and pathological remodeling (Figure 3G) (37). TKO hearts maintained robust Parkin expression under high-fat diet conditions (Figure 3H) and showed no significant induction of these pathological markers, confirming protection against diet-induced cardiomyopathy at the molecular level (Figure 3, D–G). Our analysis of mitochondrial quality control mechanisms revealed critical functional differences between WT and TKO hearts under metabolic stress. Consistent with previous studies, WT hearts subjected to high-fat diet showed paradoxical increases in baseline autophagy markers (p62 and LC3B protein levels) despite substantial Parkin reduction. This elevation of autophagy markers suggests impaired mitophagic flux and accumulation, hallmarks of dysfunctional mitochondrial quality control (Figure 3I).

Critically, these hearts lost their adaptive mitophagic response to metabolic challenge, evidenced by failure to further upregulate p62 and LC3B protein levels in mitochondrial homogenates following 24-hour fasting compared with the fed state (38, 39). This impaired dynamic response indicated that Parkin deficiency had compromised the heart’s ability to respond appropriately to metabolic stress through enhanced mitochondrial quality control (40–42). In contrast, TKO hearts maintained a markedly different and healthier mitophagic profile under high-fat diet conditions. They exhibited low baseline levels of p62, suggesting efficient basal autophagy, while preserving robust Parkin expression even under metabolic stress. Most importantly, TKO hearts retained capacity for dynamic mitophagic responses to metabolic challenge, demonstrated by appropriate increases in both LC3B and p62 levels following 24-hour fasting, responses comparable to those observed under normal chow diet conditions (Figure 3I). These findings demonstrated that sustained Parkin upregulation in TKO hearts provided comprehensive protection against both baseline mitochondrial dysfunction and loss of adaptive capacity that characterize diet-induced cardiomyopathy.

### Developmental timing of TIGAR-Parkin regulation.

**Figure 4:**
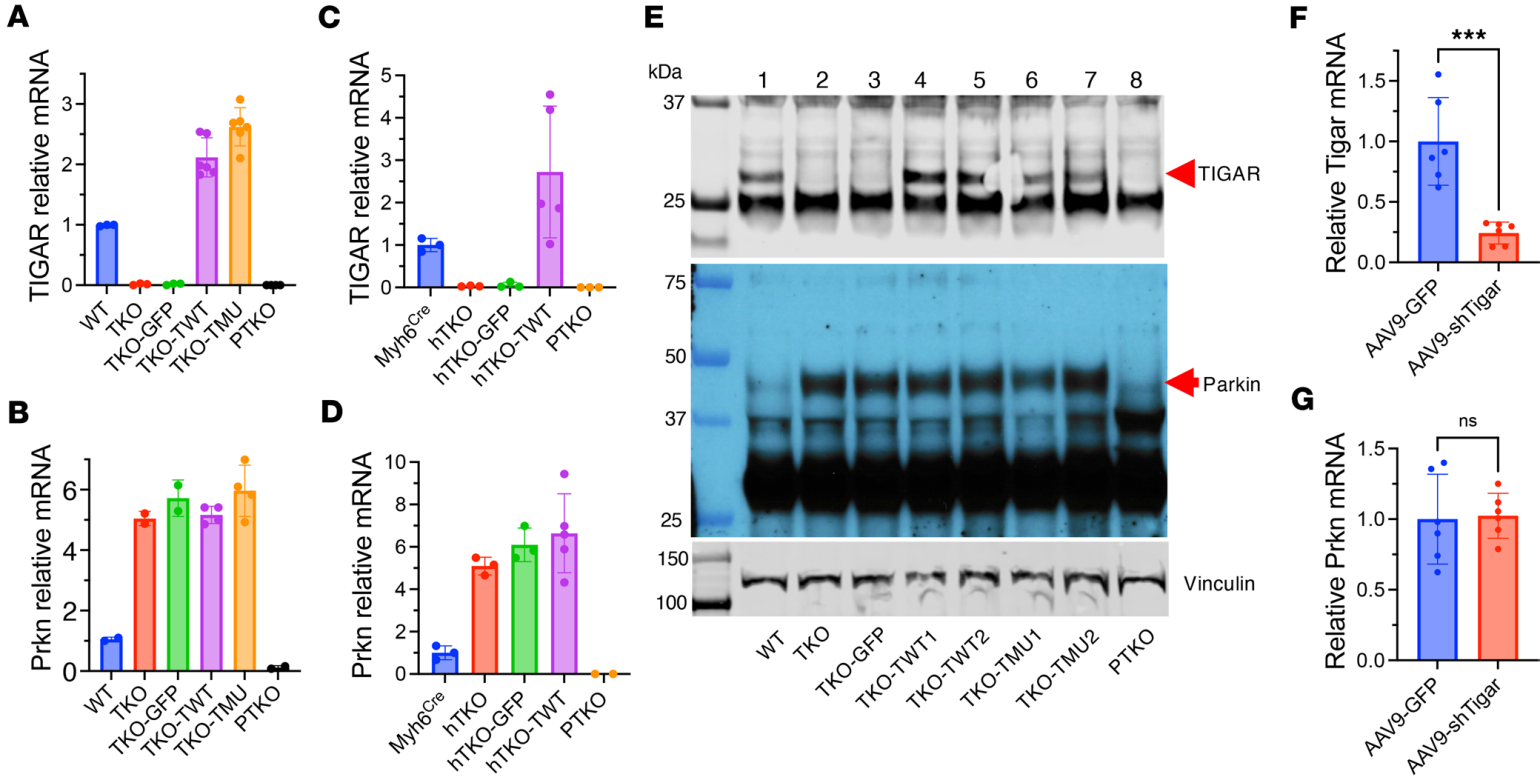
TIGAR expression in adult hearts does not directly affect Parkin expression. (**A** and **B**) qPCR analysis of TIGAR (**A**) and Parkin (**B**) mRNA in heart samples from WT and TKO mice, TKO mice injected with AAV9-cTnT-GFP control virus (TKO-GFP), AAV9-cTnT-TIGAR WT virus (TKO-TWT), or phosphatase-deficient TIGAR mutant virus (TKO-TMU), and PTKO mice. Data normalized to WT expression. (**C** and **D**) qPCR analysis of TIGAR (**C**) and Parkin (**D**) mRNA in heart samples from Myh6^Cre^ and hTKO mice, hTKO mice injected with AAV9-cTnT-GFP control virus (hTKO-GFP) or AAV9-cTnT-TIGAR WT virus (hTKO-TWT), and Parkin/TIGAR double-knockout (PTKO) mice. Data normalized to Myh6^Cre^ expression. (**E**) Western blot analysis of TIGAR (30 kDa) and Parkin (52 kDa) protein expression in heart samples from various genotypes as indicated (lanes 1–8), with vinculin (117 kDa) as loading control. Red arrows indicate TIGAR and Parkin bands. (**F** and **G**) qPCR analysis of TIGAR (**F**) and Parkin (**G**) mRNA in heart samples from WT mice treated with AAV9-GFP control or AAV9-cTnT-TIGAR shRNA. Data represent mean ± SD. Statistical significance was determined by 2-tailed Student’s *t* test. ***P < 0.005; *n* = 5 per group.

Having established that Parkin upregulation was essential for TIGAR deficiency–mediated cardioprotection, we investigated whether this relationship could be therapeutically exploited through adult interventions targeting TIGAR expression. To test whether suppressing TIGAR in adult hearts could elevate Parkin levels and recapitulate the cardioprotective phenotype, cardiac-specific adeno-associated virus 9 (AAV9) vectors encoding WT TIGAR (TWT), a phosphatase-deficient TIGAR mutant (TMU), or TIGAR-specific short hairpin RNA (shTigar) were generated for selective expression in adult cardiomyocytes. Cardiac AAV9-mediated re-expression of WT TIGAR in adult TKO mice (TKO-TWT) successfully restored TIGAR mRNA levels to approximately 2-fold above WT levels, confirming effective viral transduction and transgene expression (Figure 4A). Similarly, the phosphatase-deficient mutant achieved 2.5-fold elevation above control levels. However, despite successful TIGAR protein restoration, neither WT nor phosphatase-deficient TIGAR re-expression affected the elevated Parkin mRNA or protein levels in TKO hearts (Figure 4, B and E). This unexpected finding was reproduced in cardiomyocyte-specific knockout (hTKO) mice, in which AAV9-mediated TIGAR overexpression achieved a 2.5-fold increase in TIGAR mRNA in comparison with Myh6^Cre^ controls but failed to alter elevated Parkin expression levels characteristic of hTKO hearts (Figure 4, C and D).

Complementary experiments using AAV9-mediated cardiac-specific TIGAR silencing in adult WT mice successfully reduced TIGAR mRNA levels (76% reduction, *P* < 0.001) but were unable to induce any increase in Parkin mRNA expression (Figure 4, F and G). These findings were particularly striking given that genetic TIGAR deficiency from birth resulted in dramatic Parkin upregulation.

The failure of adult TIGAR manipulation to alter Parkin expression levels, despite successful modulation of TIGAR itself, suggested that TIGAR’s regulatory effect on Parkin occurs primarily during critical developmental windows rather than through ongoing adult regulation. Once established during development, the Parkin expression pattern appeared to become largely independent of continued TIGAR regulation. This developmental timing aligned with known biology of cardiac maturation, where the transition from fetal to adult metabolism occurs postnatally and involves major reprogramming of mitochondrial function and quality control mechanisms. The findings suggested that TIGAR deficiency during this critical period establishes persistent alterations in Parkin gene regulation that are maintained throughout adult life.

### Tissue-specific regulation and mechanistic insights.

**Figure 5:**
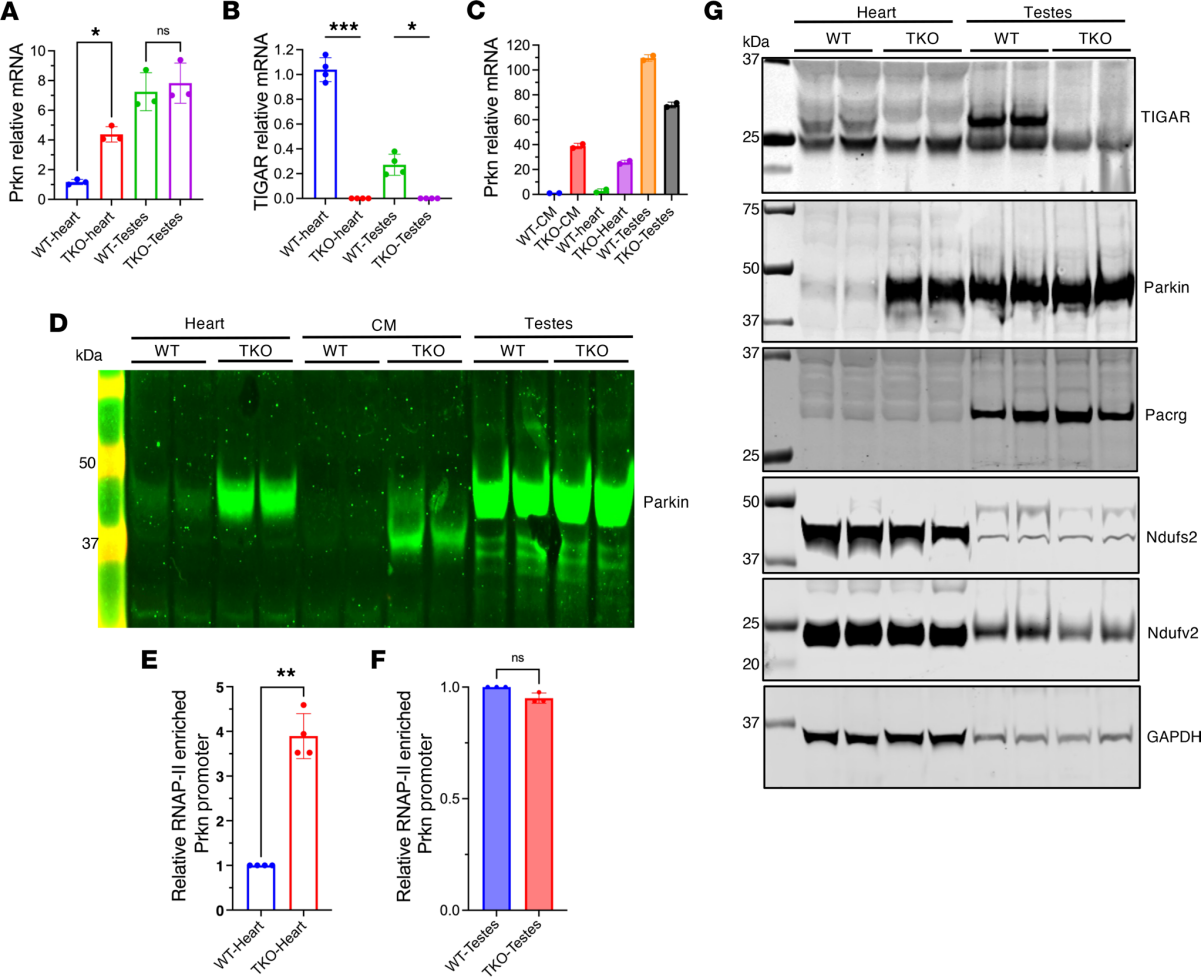
Tissue-specific regulation of TIGAR and Parkin expression in heart and testes. (**A** and **B**) qPCR analysis of Parkin (**A**) and TIGAR (**B**) mRNA levels in heart and testes from WT and TKO mice. Data normalized to WT heart expression. Data represent mean ± SD. Statistical significance was determined by 1-way ANOVA. *P < 0.05; ***P < 0.005. (**C**) qPCR analysis of Parkin mRNA levels in isolated fresh cardiomyocytes (CM), heart tissues, and testis tissues from WT and TKO mice. Data normalized to WT-CM expression. (**D**) Western blot analysis of Parkin protein levels in heart tissues, isolated cardiomyocytes (CM), and testis tissues from WT and TKO mice. (**E** and **F**) RNA polymerase II (RNAP-II) ChIP-qPCR analysis showing RNA polymerase II enrichment at the bidirectional Prkn/Pacrg promoter in WT and TKO heart tissue (**E**) and WT and TKO testis tissue (**F**). Data are normalized to WT heart or testes and represent mean ± SD. Statistical significance was determined by 2-tailed Student’s *t* test. **P < 0.01; *n* = 3–4 independent experiments. (**G**) Western blot analysis of TIGAR, Parkin, Pacrg, Ndufs2, Ndufv2, and GAPDH protein levels in heart and testes from WT and TKO mice, demonstrating tissue-specific expression patterns.

To better understand the specificity and mechanisms of TIGAR-Parkin regulation, we conducted comprehensive analyses across multiple tissue types and cellular contexts. TIGAR deficiency induced Parkin mRNA expression in heart, skeletal muscle, and brain (Figure 2, A–C) but had no effect in testicular tissue, where WT mice already expressed relatively high baseline Parkin levels (Figure 5, A and B). This tissue specificity was particularly evident when isolated cardiomyocytes were compared with testicular tissue from the same TKO animals. Isolated TKO cardiomyocytes demonstrated a dramatic 39-fold increase in Parkin mRNA expression compared with WT cardiomyocytes, while testicular tissue maintained high baseline expression in both genotypes (Figure 5, C and D). This cell-specific upregulation provided compelling evidence that enhanced Parkin expression resulted directly from TIGAR deficiency in a cell context–dependent manner.

ChIP-qPCR targeting RNA polymerase II at the Parkin promoter showed a 4-fold increase in occupancy in TKO cardiac tissue compared with WT controls (*P* < 0.0001), indicating enhanced transcriptional activation (Figure 5E). Testicular tissue showed no difference between genotypes (Figure 5F). Parkin shares a 203 bp bidirectional promoter that also controls the expression of the Pacrg gene (43). TKO hearts displayed robust Parkin protein induction but not Pacrg protein, suggesting that TIGAR’s influence involved regulatory elements beyond the shared promoter region (Figure 5G). As controls, the mitochondria respirator chain subunits Ndufs2 (NADH dehydrogenase [ubiquinone] iron-sulfur protein 2, mitochondrial) and Ndufv2 (NADH dehydrogenase [ubiquinone] flavoprotein 2, mitochondrial) and the cytosolic glycolytic protein GAPDH were unaffected in either the heart or testes of TKO mice.

### Epigenetic mechanisms of Parkin gene regulation.

**Figure 6:**
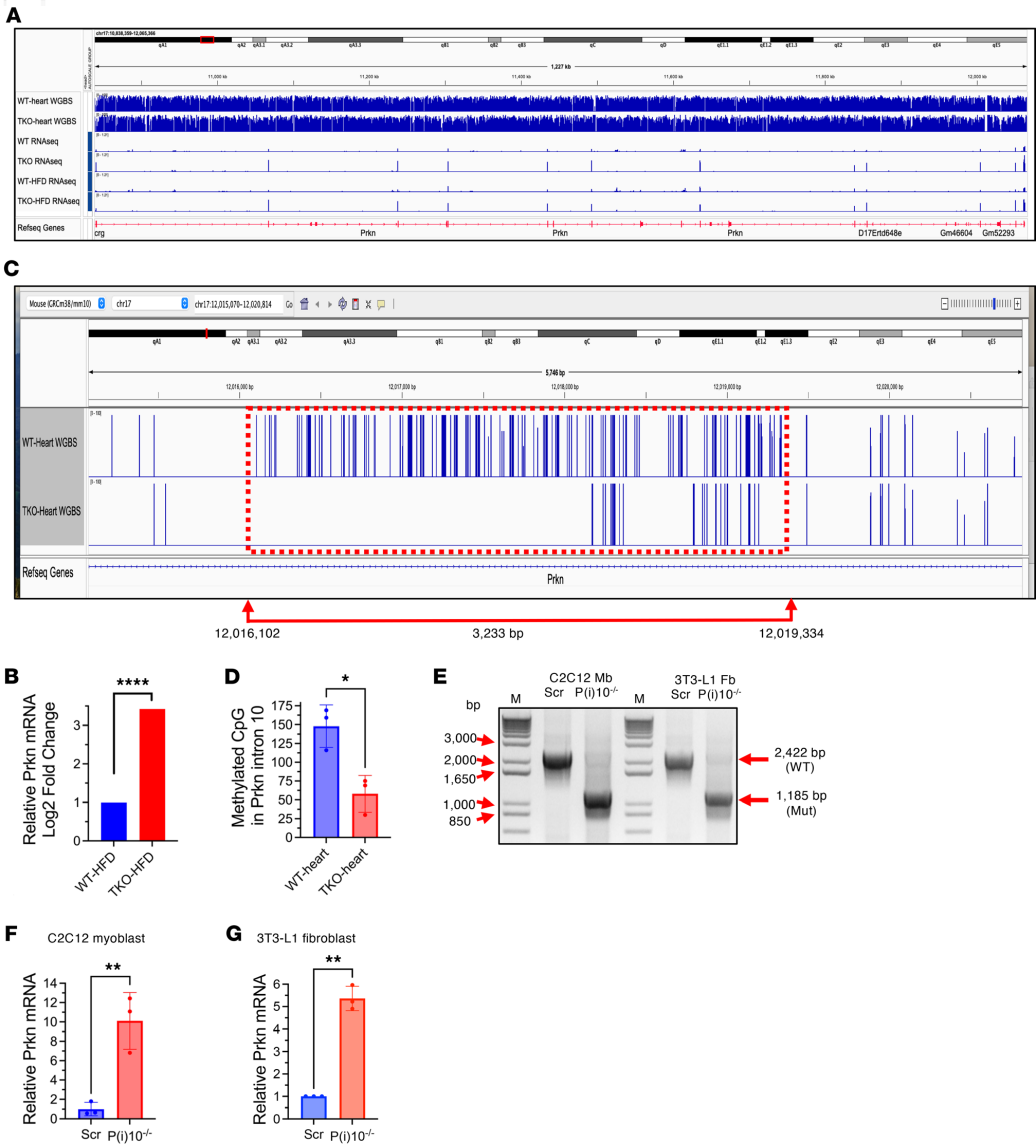
Prkn gene body methylation patterns and Parkin expression regulation. (**A** and **B**) Integrative Genomics Viewer (IGV) visualization of the Prkn gene with whole-genome bisulfite sequencing (WGBS) and RNA-seq data from WT and TKO mice hearts under normal chow and HFD conditions (**A**), with corresponding RNA-seq analysis showing significantly increased Parkin (*Prkn*) mRNA expression in TKO-HFD versus WT-HFD hearts (**B**). (**C**) Enlarged view of the differentially methylated region (DMR) within Prkn intron 10 (red dashed box, coordinates chr17:12,016,102–12,019,334; 3,233 bp). (**D**) Quantification of methylated CpG sites in the DMR in Prkn intron 10 in WT (148 ± 28) and TKO (58 ± 24) heart tissues. *P = 0.0139; *n* = 3. (**E**) CRISPR/Cas9–mediated deletion of a 14,177 bp fragment (chr17:12006677 to chr17:12020853) in Prkn intron 10 in C2C12 myoblasts (Mb) and 3T3-L1 fibroblasts (Fb). Agarose gel electrophoresis shows the predicted 1,185 bp PCR amplicon in P(i)10^–/–^ cells (gRNA/Cas9 vector transfected) versus the 2,422 bp band in scramble vector–transfected (Scr-transfected) control cells. (**F** and **G**) RT-qPCR analysis of Parkin mRNA levels in Prkn intron 10 DMR-deleted [P(i)10^–/–^] and scramble control (Scr) C2C12 myoblasts (**F**) and 3T3-L1 fibroblasts (**G**), showing 10-fold and 5-fold increases, respectively, in Parkin expression following DMR deletion. Data represent mean ± SD. Statistical significance was determined by 2-tailed Student’s *t* test. **P < 0.01; ****P < 0.0001; *n* = 3 independent experiments.

The developmental timing of TIGAR’s effects on Parkin expression, combined with persistence of elevated Parkin levels despite adult TIGAR restoration, strongly suggests developmental epigenetic mechanisms underlying this regulatory relationship. Since epigenetic histone modifications tend to be relatively reversible whereas DNA cytosine methylation (5mC) is relatively stable, we speculated that the methylation of the Parkin gene may underlie the persistent TIGAR-dependent changes in Parkin transcription. We performed comprehensive whole-genome bisulfite sequencing (WGBS) analysis of cardiac and testicular tissue from WT and TKO mice to assess DNA methylation patterns and identify differentially methylated regions (DMRs) associated with TIGAR deficiency. RNA sequencing tracks demonstrated increased Parkin transcript levels across all 12 exons in TKO hearts (Figure 6, A and B). WGBS analysis identified multiple DMRs throughout the Parkin gene body with consistently lower methylation levels in TKO hearts.

Among identified DMRs, a 3.2 kb region within Parkin intron 10 showed particularly significant hypomethylation in TKO hearts (Figure 6C). Quantitative analysis revealed that average methylation levels in this region were substantially reduced in TKO compared with WT hearts (58 ± 24 vs. 148 ± 28 methylated CpG sites, *n* = 3, *P* = 0.0139) (Figure 6D). This intronic DMR was particularly intriguing given its location within the gene body rather than the promoter region. While the Parkin promoter region contains a CpG island, no significant methylation changes were detected in this region, suggesting that TIGAR’s regulatory effects were mediated through alternative epigenetic mechanisms.

We employed CRISPR/Cas9–mediated deletion of a 14.2 kb region (chr17:12006677–12020853) encompassing the 3.2 kb DMR using dual guide RNAs (Figure 6E). PCR analysis confirmed successful deletion in both C2C12 myoblasts and 3T3-L1 fibroblasts, with mutant samples showing the expected 1,185 bp amplicon compared with the 2,422 bp product in control cells. RT-qPCR analysis demonstrated that this deletion dramatically enhanced Parkin mRNA expression in both cell types, with an approximately 10-fold increase in C2C12 myoblasts (Figure 6F) and 6-fold increase in 3T3-L1 fibroblasts (Figure 6G) compared with scramble controls. These findings provided direct functional evidence that the identified DMR normally functions as a negative regulatory element.

## Discussion

This study reveals a mechanism whereby TIGAR deficiency establishes persistent cardioprotection through developmental epigenetic programming of Parkin expression. This regulatory relationship confers broad protection against both acute ischemic injury and chronic metabolic cardiomyopathy through enhanced mitochondrial quality control mechanisms established during cardiac development and maintained throughout life.

Our discovery of TIGAR-Parkin regulatory interaction represents a significant advancement in understanding cardiac metabolic programming and mitochondrial quality control. Previous studies demonstrated cardioprotection in TIGAR-knockout mice against ischemia and pressure overload heart failure (26–28). However, the molecular mechanisms underlying this protection remained unclear. These findings reveal that TIGAR’s protective effects depend critically on developmental timing and connection to Parkin expression.

The dramatic increase in Parkin expression that we observed across multiple tissues in TIGAR-deficient mice represents one of the most striking examples of metabolic gene regulation encountered in cardiac research. The consistency of this effect across diverse tissues, combined with heart-specific knockout models, indicates that this is a fundamental regulatory relationship rather than tissue-specific adaptive responses. Genetic rescue experiments using Parkin/TIGAR double-knockout mice, which eliminated cardioprotection, provided definitive genetic evidence for Parkin’s essential role.

Our findings fundamentally challenge traditional views of diabetic and metabolic cardiomyopathy pathogenesis. Conventional understanding has emphasized excessive fatty acid uptake and increased fatty acid oxidation (lipotoxicity) as primary pathogenic mechanisms in obese diabetic hearts (44, 45). However, our results and recent findings (40, 46) demonstrate that the central pathogenic mechanism may involve impaired ATP production efficiency despite ongoing fatty acid oxidation, caused by decreased mitochondrial quality control due to lower Parkin levels that compromise mitophagy execution. This mechanistic insight explains why traditional therapeutic approaches targeting substrate utilization have shown limited clinical success — the fundamental problem lies not in substrate choice, but in the capacity of compromised mitochondrial machinery to efficiently convert substrates to ATP.

When Parkin-mediated mitophagy functions normally, as demonstrated in TKO hearts, cardiac fat accumulation and pathophysiological remodeling are prevented despite equivalent systemic metabolic stress. This finding suggests that maintaining robust mitochondrial quality control can override the deleterious effects of metabolic stress, identifying mitochondrial quality control as a more therapeutically relevant target than substrate utilization alone. The preservation of normal cardiac structure and mitophagic responses in TKO hearts under high-fat diet conditions demonstrates that sustained Parkin expression provides comprehensive protection against both baseline mitochondrial dysfunction and loss of adaptive capacity that characterize diet-induced cardiomyopathy. It is important to recognize that although we observed that TIGAR deficiency protected against the expected increase in heart mass induced by MI or diabetic cardiomyopathy, we did not directly determine whether this was a result of reduced cardiomyocyte hypertrophy, as the increase in heart mass can also result from edema (47).

In either case, the tissue-specific nature of TIGAR’s effects on Parkin expression reveals critical insights into gene regulation mechanisms. Most remarkably, testicular tissue remained completely unresponsive to TIGAR deficiency despite expressing extraordinarily high baseline Parkin levels — approximately 100-fold greater than cardiac levels. This finding suggests that testicular tissue employs fundamentally different regulatory mechanisms that bypass the methylation-dependent control observed in somatic tissues. The extremely high Parkin expression in testicular tissue may indicate that Parkin is essential for programmed and controlled cell proliferation, supported by observations that cancer tissues and immortalized cell lines typically lack Parkin expression. The complete bypass of TIGAR-mediated regulation in germline tissue further supports the concept that different tissues employ distinct epigenetic regulatory mechanisms for the same gene.

One of the most striking aspects of our findings relates to how developmental programming shapes cardiovascular health throughout life. The concept that cardiac metabolism during early developmental stages programs lifelong cardiac function through epigenetic mechanisms represents a paradigm shift in understanding cardiovascular disease susceptibility. The fact that adult TIGAR manipulation could not alter Parkin expression levels, despite successfully changing TIGAR itself, demonstrates that this regulatory relationship becomes established during critical developmental windows and remains fixed in adult tissues.

This developmental timing aligns with the well-characterized postnatal shift in cardiac metabolism from glycolysis-dependent fetal metabolism to fatty acid oxidation–dependent adult metabolism (19). During this transition, cardiomyocytes undergo massive mitochondrial expansion, maturation of respiratory complexes, and establishment of quality control mechanisms that serve the heart throughout adult life (5, 18, 19). Our findings suggest that metabolic conditions during critical developmental periods can permanently alter expression of key mitochondrial quality control genes through epigenetic mechanisms, with profound implications for understanding how early environmental influences affect lifelong cardiovascular health. These discoveries highlight the critical importance of optimal maternal and childhood nutrition in programming cardiac resilience, suggesting that nutritional interventions during early development could establish lifelong protection against cardiovascular disease through enhanced mitochondrial quality control pathways.

Our identification of a specific 3.2 kb differentially methylated region within Parkin intron 10 that controls gene expression provides important mechanistic insights and potential therapeutic targets. Unlike promoter-proximal regulatory elements that control transcriptional initiation (48), this intronic region likely influences transcriptional elongation efficiency through the large Parkin gene. Functional validation of this regulatory element through CRISPR-mediated deletion, which enhanced Parkin expression 6- to 10-fold in multiple cell types, demonstrates its potential as a therapeutic target. Future approaches might involve targeted demethylation of this region using emerging epigenome editing technologies, potentially allowing manipulation of Parkin expression levels in adults.

These findings have several important clinical implications extending beyond basic mechanistic insights. The remarkable preservation of cardiac function following MI in TIGAR-deficient mice (maintaining 60% vs. 36% of baseline function) represents a degree of cardioprotection that would be clinically transformative if achievable in human patients. Enhanced survival of cardiomyocytes within damaged zones, demonstrated by preservation of cardiac troponin I, SERCA2a, and other essential proteins, suggests that enhanced Parkin expression could extend the window for reperfusion therapy and improve outcomes even when treatment is delayed (49, 50).

The complete resistance to diet-induced cardiac dysfunction observed in TKO mice, despite equivalent weight gain, directly addresses one of the major challenges in managing diabetic cardiovascular disease. Current therapies often fail to prevent cardiac complications even with optimal glycemic control, highlighting the need for approaches targeting underlying mitochondrial dysfunction (8, 51). The preservation of normal cardiac structure and mitophagic responses in TKO hearts suggests that enhancing Parkin expression could prevent diabetic heart disease development (40–42).

The dysfunction observed in WT mice on high-fat diet closely mirrors the clinical syndrome of heart failure with preserved ejection fraction, which represents approximately half of all heart failure cases and currently lacks effective therapies (52). The protection against this condition in TKO mice suggests that targeting mitochondrial quality control could provide new therapeutic approaches for this challenging clinical problem.

Our study provides important insights into mitochondrial quality control regulation and function in the heart. The enhanced mitophagic responses observed in TKO hearts during metabolic stress, combined with preservation of cytosolic Parkin reserves even under prolonged stress conditions, demonstrate that sustained high-level Parkin expression enhances cardiac adaptive capacity without compromising baseline mitochondrial function. The preservation of normal mitochondrial protein levels under baseline conditions, despite enhanced mitophagic machinery, indicates that upregulated Parkin does not result in excessive mitochondrial degradation. This suggests that mitophagy is appropriately regulated and responds primarily to damaged or dysfunctional organelles rather than randomly targeting healthy mitochondria.

The loss of adaptive mitophagic responses in WT hearts subjected to high-fat diet, demonstrated by failure to upregulate autophagy markers during fasting despite metabolic stress, highlights how mitochondrial quality control becomes progressively impaired in metabolic disease (53). The preservation of these responses in TKO hearts demonstrates that enhanced Parkin expression can prevent this deterioration and maintain cardiac adaptive capacity under adverse conditions (54).

Our findings complement and extend recent studies using pharmacological Parkin activators such as PR-364, which demonstrated cardioprotection following MI (16). The preservation of mitochondrial gene expression in TKO infarct zones closely matched observations with PR-364 treatment, suggesting common mechanisms involving enhanced mitochondrial quality control. However, the genetic approach provides several advantages over pharmacological intervention. First, it establishes that beneficial effects result specifically from enhanced Parkin expression rather than off-target drug effects. Second, it demonstrates that sustained elevation of Parkin expression is well tolerated and beneficial throughout the lifespan. Third, it reveals the importance of developmental timing in establishing effective cardioprotection.

These investigations were conducted in male mouse models; we do not know whether the functional and mechanistic protection effect of TIGAR deficiency will also occur in female mice. In addition, the translation to human physiology will also require validation in human tissues and clinical studies. Additionally, optimal methods for therapeutic intervention targeting this pathway remain to be determined, and potential long-term consequences of sustained Parkin elevation require additional investigation. Future studies should focus on developing approaches to target our identified epigenetic regulatory elements in adult male and female animals, investigating precise metabolic pathways linking TIGAR deficiency to DNA methylation changes, and assessing relevance of these findings in human populations.

Nevertheless, the concept of developmental programming revealed by this study has broader implications for cardiovascular medicine beyond the specific TIGAR-Parkin connection. It suggests that cardiac responses to adult stressors are fundamentally shaped by developmental experiences and that interventions during critical developmental windows could have lifelong beneficial effects. This concept is particularly relevant given growing recognition that cardiovascular disease often begins early in life, even when clinical manifestations appear decades later (55, 56). These findings suggest that identifying and targeting developmental programming mechanisms could provide new approaches for preventing cardiovascular disease before it begins. Furthermore, the identification of specific epigenetic regulatory elements provides a framework for understanding how environmental factors during development can permanently alter disease susceptibility. This knowledge could inform recommendations for maternal and early childhood health interventions aimed at optimizing cardiovascular development.

Our study identifies a TIGAR-Parkin regulatory pathway operating through epigenetic mechanisms during cardiac development to establish lifelong cardioprotection. TIGAR deficiency enhances Parkin expression through reduced methylation of a specific regulatory region in the Parkin gene, resulting in improved mitochondrial quality control and enhanced cardiac resilience against both MI and metabolic stress. These findings provide new therapeutic targets for cardiovascular disease and highlight how developmental programming mechanisms determine lifelong cardiovascular health, opening new avenues for both prevention and treatment of heart disease.

## Methods

### Sex as a biological variable

This study exclusively examined male mice to reduce variability associated with sex-dependent differences in cardiac physiology and metabolic responses in the models used. It is unknown whether the findings are fully generalizable to female mice. However, increased Parkin expression was also observed in female mouse hearts.

### Animal studies and tissue collection

Mice were housed in a facility with a 12-hour-light/dark cycle and maintained on normal chow diet (5053, LabDiet) containing 62.3% carbohydrates, 24.5% protein, and 13.1% fat by caloric content, or a high-fat diet (HFD; D12492, Research Diets) containing 60% fat, 20% carbohydrates, and 20% protein by caloric content. TKO and Tigar^fl/fl^ mice were produced as described in our previous paper (30). The Tigar^fl/fl^ mice were generated from C57BL/6N-Tigar^tm1a(EUCOMM)Wtsi/Wtsi^ mice obtained from the Wellcome Trust Sanger Institute (Hinxton, United Kingdom) (30). Heart cardiomyocyte–specific Tigar-knockout (hTKO) mice were produced by crossing of Tigar^fl/fl^ mice with cardiac-specific α-myosin heavy chain–Cre (Myh6^Cre^) mice (stock 011038, The Jackson Laboratory). Genotyping was performed using primers 9543, 9544, oIMR8744, and oIMR8745 for Myh6^Cre^- and Tigar^fl/fl^-specific primers (30) for hTKO mice. Parkin-knockout (PKO) mice were purchased from The Jackson Laboratory (stock 006582). Parkin and Tigar double-knockout (PTKO) mice were produced by crossing of PKO with TKO. At 16 weeks of age, mice underwent left anterior descending coronary artery ligation and echocardiographic imaging.

### Mouse heart tissue collection and processing for genomic DNA, total RNA, 

### and protein isolation

Adult mice were briefly anesthetized in an induction chamber with 3%–4% vaporized isoflurane (VetEquip) until loss of righting reflex, followed by cervical dislocation following institutional guidelines and approved protocols. Hearts were rapidly excised via thoracotomy and immediately placed in ice-cold phosphate-buffered saline (PBS; pH 7.4) to remove residual blood. The atria were carefully removed and the ventricles quickly blotted dry with lint-free tissue paper.

The ventricles were immediately snap-frozen using a cryogenic tissue clamp pre-chilled in liquid nitrogen. Frozen cardiac tissue was then transferred to a mortar immersed in liquid nitrogen and homogenized to a fine powder with a pre-chilled pestle, maintaining cryogenic conditions throughout the pulverization process to prevent tissue thawing. The tissue powder was collected into pre-labeled, sterile cryovials and stored at –80°C until further analysis.

### Total RNA extraction and RT-qPCR

Total RNA isolation and RT-PCR were performed as previously described (30). Relative mRNA expression was calculated using ΔΔCt method and normalized to RNA18S1 or Rplp0. The TaqMan primer-probe assays used in this study included *Tigar* (TP53-induced glycolysis and apoptosis regulator; assay ID Mm00621530_m1), *Tgfb2* (transforming growth factor-β2; Mm00436955_m1), *Tnnt2* (cardiac troponin T; Mm01290256_m1), and *Prkn* (Parkin; Mm01323528_m1). *RNA18S1* (eukaryotic 18S rRNA; 4310893E) and *Rplp0* (ribosomal protein, large, P0; Mm00725448_s1) were used as endogenous controls.

For detection of mutant TIGAR (H11A/E102A/H198A) expression, custom primer-probe sets were used. The Tigar primer-probe set spanning exons 3–4 (PrimeTime Std qPCR Assay, Mm.PT.56a.16927616, 9630033F20Rik, Integrated DNA Technologies [IDT]) and the internal control *Rplp0* primer-probe set spanning exons 5–6 (Mm.PT.58.43894205) were used with PrimeTime Gene Expression Master Mix (catalog 1055772, IDT) following the manufacturer’s protocol.

### Immunoblotting

Samples were prepared from cultured cells (washed with cold PBS) or tissues by homogenization with radioimmunoprecipitation assay (RIPA) lysis buffer (sc-24948, Santa Cruz Biotechnology) containing Halt protease and phosphatase inhibitor mixture (catalog 78442, Thermo Fisher Scientific), 20 μM MG132, and 20 μM ALLN (EMD Millipore) using ceria-stabilized zirconium oxide beads (MidSci). Homogenates were centrifuged for 15 minutes at 21,000*g* at 4°C and supernatants collected for protein quantification using the BCA method. Protein samples were separated by either self-cast SDS-PAGE or SurePAGE (Bis-Tris) precast gel (GenScript) and transferred to nitrocellulose membrane using iBlot 2 Blotting System (Thermo Fisher Scientific). The immunoblot membrane was blocked with Pierce Protein-Free T20 (TBS) blocking buffer (product 37571, Thermo Fisher Scientific) or 6% milk in TBS with Tween 20 (TBST), then incubated with the primary antibody in blocking buffer or 1% milk TBST. Blots were washed in TBST and incubated with either IRDye 800CW secondary antibody (LI-COR) or horseradish peroxidase–conjugated secondary antibody in blocking buffer. The membrane was washed with TBST and visualized using either the Odyssey CLx Imaging System (LI-COR) or enhanced chemiluminescence (Pierce, Thermo Fisher Scientific) method. Commercial primary antibodies were purchased from the following sources: TIGAR (sc-677290), SERCA2 (sc-376235), Parkin (sc-32282), Tom70 (sc-390545), NDuFS2 (sc-390596), NDuFV2 (sc-271620), vinculin (sc-73614), SDHA (sc-166909, HRP), SQSTM1/p62 (sc-48402), MAP LC3B (sc-376404), and β-actin (sc-47778) from Santa Cruz Biotechnology; Parkin (2132S), ParkinSer65 (36866S), troponin I (13083S), VDAC (4866S), ubiquitin (43124S), and α-actinin-1 (6487S) from Cell Signaling Technology; Parkin (702785) and PACRG (PA5-110069) from Thermo Fisher Scientific; complex I 75 kDa (ABN302) from EMD Millipore; and GAPDH (MBL-M171-3) from Cosmo Bio USA.

### Chromatin immunoprecipitation and quantitative PCR analysis

Left ventricular tissue (approximately 40 mg) was collected from WT and TKO mouse hearts, washed with ice-cold PBS to remove blood, and immediately minced on an ice-embedded Petri dish using a pre-chilled razor blade. The minced tissue was cross-linked with 1% formaldehyde (28908, Thermo Fisher Scientific) and processed for chromatin isolation using the ChromaFlash Chromatin Extraction Kit (P-2001, EpigenTek). Chromatin samples (300 μL per 1.5 mL tube) were sonicated using a Diagenode Bioruptor for 15 cycles (30 seconds on/30 seconds off) at high-power setting to generate DNA fragments ranging from 150 to 1,000 bp. Chromatin immunoprecipitation (ChIP) was performed using the ChromaFlash One-Step ChIP Kit (P-2025, EpigenTek) following the manufacturer’s instructions. DNA-protein complexes were immunoprecipitated using anti–RNA polymerase II monoclonal antibody (CTD4H8; A-2032-100, EpigenTek), with non-immune IgG (P-2025, EpigenTek) serving as a negative control. The enriched DNA fragments and input DNA (10% of sample chromatin) were purified, released, and eluted. DNA concentration was determined using the Qubit dsDNA High Sensitivity Quantification Assay (Thermo Fisher Scientific). Quantitative PCR analysis was performed using primers targeting the Prkn/Pacrg bidirectional promoter (forward: GTCAACATTAGGAGACGCTAGTC; reverse: GCAACTGTCTTCGCTGGTA) to generate a 76 bp amplicon, with mouse RPL30 intron 2 primers (7015P, Cell Signaling Technology) serving as an internal control. PCR reactions were conducted using PowerUp SYBR Green Master Mix (A25741, Thermo Fisher Scientific) on a QuantStudio 6 Flex platform. ChIP-qPCR data were analyzed using the 2^–ΔΔCt^ method.

### MI model and cardiac function assessment

Male mice (16 weeks old) underwent permanent ligation of the left anterior descending (LAD) coronary artery to induce MI (57, 58). After baseline cardiac assessment, a left thoracotomy (1.5 cm) was performed through the fourth intercostal space under appropriate anesthesia. After retraction of the lungs and opening of the pericardium to expose the heart, the LAD coronary artery was identified and ligated at its proximal region using 8-0 silk suture. The ligation site was positioned between the pulmonary outflow tract and left atrial edge, approximately 2 mm distal to the left auricle tip. Successful arterial occlusion was confirmed by visible pallor of the anterior left ventricular wall. Postoperative care included maintaining body temperature using a heating pad until full recovery. Sham-operated controls underwent identical surgical procedures without arterial ligation.

Cardiac function was evaluated via transthoracic 2-dimensional echocardiography using a 12 MHz probe (VeVo, Visualsonics) at baseline and weeks 1, 2, and 4 after surgeries. Left ventricular parameters were measured through M-mode imaging in the parasternal short-axis view, including end-diastolic and end-systolic dimensions and septal and posterior wall thicknesses, to calculate fractional shortening and ejection fraction.

### Adult mouse cardiomyocyte isolation

Cardiomyocytes were isolated as previously described (57, 59). Adult mice were injected intraperitoneally with 2,000 IU/kg heparin 5 minutes before heart isolation. Hearts were isolated and perfused using a Langendorff apparatus. Hearts were initially retroperfused for 20 minutes with calcium-free cardiac cell isolation buffer (containing in mM: NaCl, 113; KCl, 4.7; NaH_2_PO_4_, 0.6; KH_2_PO_4_, 0.6; HEPES, 10; glucose, 7; taurine, 15; 2,3-butanedione monoxime, 10; MgSO_4_, 1.2; pH 7.4) supplemented with 5 mM EDTA at 37°C. This was followed by enzymatic digestion through retroperfusion with the same buffer containing 300 U/mL collagenase type 4 (CLS-4, Worthington Biochemical Corp.) and 1.2 U/mL protease from *Streptomyces griseus* type XIV (P5147, MilliporeSigma) for 30 minutes.

After perfusion, the left ventricle was carefully dissected and minced into approximately 1 mm^3^ pieces in cardiac cell isolation buffer containing collagenase II (300 U/mL) and protease XIV, then gently dissociated using forceps. The enzymatic digestion was terminated after 10 minutes by addition of cardiac cell isolation buffer supplemented with 5% sterile, exosome-free FBS. The cell suspension was filtered through a 100-μm-pore-size nylon mesh filter (22363549, Fisherbrand) to remove undigested tissue.

Isolated cells were allowed to settle by gravity in 15 mL tubes for approximately 20 minutes. During this sedimentation period, calcium was gradually reintroduced in 4 sequential steps (at 4-minute intervals) to reach final concentrations of 0.06, 0.24, 0.6, and 1.2 mM, respectively. Rod-shaped cardiomyocytes settled to form a pellet at the bottom of the tube and were collected for subsequent experiments. The quality and morphology of isolated cardiomyocytes were confirmed by microscopic examination.

Cardiomyocyte total RNA was extracted using Quick-RNA Miniprep (R1055, Zymo Research Inc.) for *Prkn* mRNA quantification by qPCR. Total protein of cardiomyocytes was isolated using the Total Protein Extraction Kit for Animal Cultured Cells and Tissues (SD-001/SN-002, Invent Biotechnologies Inc.), followed by Western blot analysis of Parkin protein expression as described previously.

### Tissue collection for RNA, protein, and immunofluorescence analyses after LAD-MI

For RNA and protein analyses, the infarct, border, and remote zones were isolated, immediately snap-frozen in liquid nitrogen, pulverized in liquid nitrogen, and stored at –80°C for subsequent RNA and protein extraction. For immunofluorescence studies, fresh cardiac tissues were embedded in Tissue-Tek OCT compound and sectioned to 5 μm thickness. Immunostaining was performed using anti-periostin (PA5-34641, Invitrogen) and anti–α-actinin (sc-17829, AF488, Santa Cruz Biotechnology) antibodies. Stained sections were visualized and images captured using an Echo Revolve Microscope.

### Subcellular fractionation of mouse heart tissue for isolation of cytosolic and mitochondrial components

Mouse hearts were dissected from isoflurane-anesthetized animals and washed with ice-cold mitochondria assay solution (MAS; 70 mM sucrose, 220 mM d-mannitol, 5 mM KH_2_PO_4_, 5 mM MgCl_2_, 1 mM EGTA, 2 mM HEPES, 0.025% fatty acid–free BSA, pH 7.4, adjusted with KOH) to remove blood. The heart was placed on an ice-embedded Petri dish and finely minced using a pre-chilled razor blade. The minced heart tissue was homogenized in cold MAS buffer with Halt protease and phosphatase inhibitor mixture (catalog 78442, Thermo Fisher Scientific), 20 μM MG132, 20 μM ALLN (MilliporeSigma) using 25 strokes in a glass-glass Dounce homogenizer, followed by centrifugation at 1,000*g*, 4°C for 5 minutes. The supernatant was further centrifuged at 10,000*g*, 4°C for 15 minutes. The supernatant was concentrated using Amicon Ultra-0.5 Centrifugal Filter Units (UFC500324, MilliporeSigma) and immediately stored at –80°C as the cytosolic fraction. The pellet was resuspended in RIPA lysis buffer (sc-24948, Santa Cruz Biotechnology) containing Halt protease and phosphatase inhibitor mixture, 20 μM MG132, 20 μM ALLN, 1% SDS, and 1% *N*-lauryl sarcosine sodium salt as the mitochondrial fraction. The protein concentration of both fractions was determined using a Pierce BCA Protein Assay Kit (Thermo Fisher Scientific).

### RNA sequencing and transcriptomic analysis

Total RNA was isolated from heart tissues using the Direct-zol RNA Miniprep Plus kit (R2073, Zymo Research Inc.) following the manufacturer’s instructions. For post-MI analysis, RNA was extracted from distinct cardiac regions (infarct, remote, and boundary zones) following LAD artery ligation. For diet studies, RNA was isolated from whole hearts of mice fed either standard chow or HFD for 6 months.

RNA sequencing was performed by 2 independent service providers. Post-MI heart tissue samples were submitted to GENEWIZ (Azenta Life Sciences) for library preparation and sequencing. Raw RNA-seq reads were first subjected to quality control and adapter trimming using Trim Galore (v0.6.5; https://doi.org/10.5281/zenodo.5127899). Transcript quantification was performed using Kallisto (v0.46) with mm10 reference transcriptome (60). The transcript-level abundance estimates were summarized to gene-level counts using the tximport package in R. Differential expression analysis was conducted using the edgeR package (v3.34.1).

For diet studies, total RNA from chow diet and HFD groups from WT and TKO mice was processed. For each experimental group, heart tissues from 2 mice (50 mg/mouse) were pulverized and pooled, with 3 pooled biological replicates per condition. These samples were submitted to NovaGen Biotech Labs Inc. for library preparation and RNA sequencing on an Illumina NovaSeq X Plus platform. Raw RNA-seq reads were processed using Trim Galore (v0.6.7) to remove adapter sequences and low-quality bases. Transcript-level quantification was performed using Kallisto (v0.46.2) with mm10 reference transcriptome, and transcript abundance estimates were imported into R using the tximport package to generate gene-level count matrices. Differential expression analysis was conducted using DESeq2 (v1.32.0).

For genome browser visualization, the trimmed reads from the diet studies dataset were aligned to the reference genome using HISAT2 (v2.2.1) (61) with default parameters. Aligned reads were sorted and indexed using SAMtools (62), and normalized coverage tracks (bigWig files) were generated using bamCoverage from deepTools (v3.5.1) (63), using counts per million mapped reads (CPM) normalization. The tracks were visualized using the Integrative Genomics Viewer (IGV) (64).

### Whole-genome bisulfite sequencing and DNA methylation analysis

High–molecular weight genomic DNA was isolated from heart and testis tissues using the Monarch HMW DNA Extraction Kit (T3060L, New England BioLabs) following the manufacturer’s instructions. Whole-genome bisulfite sequencing (WGBS) was performed by 2 independent service providers using distinct sample sets.

In the first analysis, purified WT and TKO cardiac genomic DNA samples were submitted to Azenta Life Sciences for WGBS. The NEBNext Enzymatic Methyl-seq Kit was used for sample preparation following Azenta’s established protocols. Raw reads were first trimmed using Trim Galore (v0.6.7), with options “--adapter AGATCGGAAGAGCACACGTCTGAACTCCAGTCA --adapter2 AGATCGGAAGAGCGTCGTGTAGGGAAAGAGTGT --length 15 --clip_r1 8 --clip_r2 8 --three_prime_clip_r1 8 --three_prime_clip_r2 8”. The trimmed reads were then aligned to bisulfite-converted mm10 reference genome using Bismark (v0.23.1) (65) with Bowtie 2 (v2.4.5) (66) as the underlying alignment algorithm. After alignment, duplicates were removed using deduplicate_bismark. Methylation calls were then extracted using bismark_methylation_extractor, which generated BedGraph files reporting methylation levels at single-base resolution. These de-duplicated BedGraph files were converted to TDF (Tiled Data Format) files using IGVtools (v2.7.2) for visualization of DNA methylation patterns in the Integrative Genomics Viewer (IGV).

In the second analysis, high–molecular weight genomic DNA was isolated from both heart tissues (*n* = 2 for each tissue type per genotype) from WT and TKO mice. These samples were submitted to Novogene for WGBS, library preparation, and sequencing using their established protocols. Raw reads were trimmed using Trim Galore (v0.6.7), with options “--adapter AGATCGGAAGAGCGTCGTGTAGGGAAAGAGTGTAGATCTCGGTGGTCGCCGTATCATT --adapter2 GATCGGAAGAGCACACGTCTGAACTCCAGTCACGGATGACTATCTCGTATGCCGTCTTCTGCTTG --length 15 --clip_r1 10 --clip_r2 15 --three_prime_clip_r1 10 --three_prime_clip_r2 10”. After trimming, the same bioinformatics pipeline was applied as described above, including alignment with Bismark, duplicate removal, methylation call extraction, and visualization preparation for IGV analysis.

### CRISPR/Cas9–mediated deletion of mouse Parkin intron 10 differentially methylated region

The identified differentially methylated region (DMR) within intron 10 of the mouse Parkin (Prkn) gene was targeted for deletion. Guide RNA (gRNA) sequences targeting both ends of the DMR were selected using CRISPR Target Track Setting (UCSC Genome Browser), with the left and right gRNA sequences corresponding to chromosomal positions chr17:12006677–12006699 and chr17:12020834–12020856, respectively. A dual gRNA mammalian CRISPR vector system was designed and synthesized by VectorBuilder Inc., including both the Prkn intron 10 DMR targeting construct (pRP[2CRISPR]-EGFP/Puro-hCas9-U6>(Prkn Chr17:12006677–12006699)-U6>(Prkn Chr:12010834–12020856)) and a scrambled gRNA control vector (pRP[CRISPR]-EGFP/Puro-hCas9-U6>Scramble_gRNA1). Sequence verification was performed by the manufacturer using Sanger sequencing. The targeting plasmid expresses Cas9 nuclease with two gRNAs designed to delete a 14,180 bp region within intron 10 of the Parkin gene. VectorBuilder prepared the plasmids by maxiprep purification, yielding greater than 1 μg/μL concentration (300 μL total volume).

Both VectorBuilder-produced plasmids contained EGFP and puromycin resistance markers for cellular selection and transfection tracking. 3T3-L1 fibroblast cells (CL-173, ATCC) or C2C12 myoblast cells (CRL-1772, ATCC) were seeded in 10 cm plates at approximately 50% confluence and maintained in antibiotics-free 10% FBS DMEM. Cells were transfected with 15 μg of plasmid DNA diluted in 0.75 mL Opti-MEM I (1×; catalog 31985062, Thermo Fisher Scientific) using 60 μL EndoFectin Max Transfection Reagent (GeneCopoeia), which was also diluted in 0.75 mL Opti-MEM, following the manufacturer’s protocol. Puromycin selection (catalog code ant-pr-1, InvivoGen) was initiated 24 hours after transfection and maintained for 72 hours. The selected cells were subsequently maintained in 10% FBS DMEM.

Genomic DNA was isolated using the GeneJet Genomic DNA Purification Kit (K0721, Thermo Fisher Scientific). PCR validation of the deletion was performed using 3 primers, which differentiate WT and mutant DNAs. The forward primer sequence was 5′-GACAGTGGTCCTAAACACTATTGTGG-3′, corresponding to positions 12,006,286–12,006,311. The WT reverse primer (5′-GCTGTTATGTTAGGTTTAGCAGGGAA-3′; 12,008,682–12,008,707) generated a 2,422 bp amplicon, whereas the mutant-specific reverse primer (5′-TACAGTGGACTCCAACGCAGTA-3′; 12,021,629–12,021,650) produced a 1,185 bp amplicon. PCR amplification was performed using GoTaq Master Mixes 2X (M7123, Promega).

Cycling conditions for amplifying longer PCR products were used as described on QIAGEN’s website (Bench Guide, PCR, Amplification of long PCR products): 95°C for 2 minutes, followed by 40 cycles of denaturation at 94°C for 10 seconds, annealing at 55°C for 60 seconds, and extension at 68°C for 90 seconds, with a final hold at 4°C.

Successful deletion yielded a 1,185 bp product compared with the 2,422 bp WT product when resolved on 1.0% agarose gel made in 0.5× Tris-borate-EDTA (TBE) buffer. To assess the impact of DMR deletion on Parkin gene expression, RNA was extracted using Quick-RNA Miniprep (R1055, Zymo Research Inc.) for *Prkn* mRNA quantification by qPCR.

### AAV9 vector construction and viral production

Two distinct AAV9 vector systems with cardiac-specific expression were outsourced for production:

#### AAV9-cTnT-Tigar vectors.

Custom AAV9 vectors expressing WT *Tigar* (NM_177003) and a phosphatase-deficient Tigar mutant were designed and manufactured by Charles River Laboratories for cardiac-specific gene delivery. The coding sequences (895 bp) were subcloned into a pAV9-cTnT-GFP backbone containing an IRES-GFP reporter. For the phosphatase-deficient mutant, 3 critical catalytic residues (His11, Glu102, and His198) were simultaneously substituted with alanine residues, thereby abolishing phosphatase activity while maintaining protein expression. Charles River verified the resulting constructs (pAV9-cTnT-NM_177003-IRES-GFP and pAV9-cTnT-NM_177003mutant-IRES-GFP) by full-length sequencing and performed viral packaging, yielding high-titer preparations (more than 1 × 10^13^ genomic copies/mL) delivered as 4 × 250 μL aliquots per construct. Control AAV9 viral particles containing only GFP (pAV9-cTnT-GFP, catalog CV17196-AV9) were purchased from Charles River.

#### AAV9-cTnT-Tigar-shRNA vectors.

For Tigar knockdown studies, an shRNA construct (pAAV[3miR30]-cTnT>EGFP:mTigarshRNA#1,2,3:WPRE) was designed and generated by VectorBuilder Inc. A corresponding control vector with a scrambled shRNA sequence (pAAV[miR30]-cTnT>EGFP:Scramble[miR30-shRNA#1]:WPRE) was also constructed by VectorBuilder. The company packaged both vectors into AAV9 particles through medium-scale production followed by ultrapurification, yielding titers exceeding 3.65 × 10^13^ genomic copies/mL, delivered as 10 × 50 μL aliquots per vector.

### In vivo delivery

For AAV9 administration, the commercially produced viral preparations were diluted to 1.2 × 10^10^ viral particles per microliter in sterile physiological saline. Mice were anesthetized with isoflurane, and 50 μL of the diluted viral suspension (total: 6 × 10^11^ viral particles per mouse) was administered via retro-orbital injection. Animals were monitored after injection for adverse events, and cardiac transgene expression was assessed 4 weeks after administration.

### Statistics

GraphPad Prism 10 (GraphPad Software) was used for data processing, analyses, and graph production in the experiments. The number of independent experimental replications and the average with standard deviation are provided in the figure legends. Unpaired 2-tailed Student’s *t* tests or non-parametric tests (Mann-Whitney, Kruskal-Wallis) were used for statistical analyses. *P* values of less than 0.05 were considered significant.

### Study approval

All animal procedures were performed in accordance with protocols approved by the Albert Einstein College of Medicine Institutional Animal Care and Use Committee.

### Data availability

RNA sequencing and WGBS data generated in this study, including associated DNA methylation analyses, were deposited in the Gene Expression Omnibus (GEO) database under the following accession numbers: GSE317048 (Transcriptomic profiling of WT and TKO mouse hearts); GSE317050 (RNA-seq analysis of Parkin expression in WT and TKO mouse hearts under normal chow and HFD conditions); GSE317052 (DNA methylation analysis of the Parkin gene in WT and TKO mouse heart); and GSE317054 (DNA methylation analysis of the Parkin gene in WT and TKO mouse heart). Supporting data values associated with the main article and supplemental material, including values for all data points shown in graphs and values underlying reported means, are provided in the Supporting Data Values file. Additional data and analytic code are available upon reasonable request.

## Author contributions

YT designed research studies, conducted experiments, acquired data, analyzed data, provided reagents, and wrote the manuscript. SSJ conducted experiments, acquired data, analyzed data, and provided reagents. LL acquired data, analyzed data, and wrote the manuscript. XW conducted experiments, acquired data, and provided reagents. AMX conducted experiments, acquired data, analyzed data, and provided reagents. FY designed research studies and acquired funding. DF contributed to manuscript editing and revision. GS and JEP supervised the study, designed research studies, acquired data, analyzed data, provided reagents, wrote the manuscript, and acquired funding.

## Conflict of interest

The authors have declared that no conflict of interest exists.

## Funding support

This work is the result of NIH funding, in whole or in part, and is subject to the NIH Public Access Policy. Through acceptance of this federal funding, the NIH has been given a right to make the work publicly available in PubMed Central.

- NIH grants DK033823 and DK020541 (to JEP) and HL146691 (to GS).

## Supplementary Material

Supplemental data

Unedited blot and gel images

Supporting data values

## Version 1 02/26/2026

In-Press Preview

## Version 2 04/22/2026

Electronic publication
