# Supplementary material for: TIGAR deficiency enhances cardiac resilience through epigenetic programming of Parkin expression
Source: JCI Insight. 2026 Feb 26;11(8):e200105. doi: 10.1172/jci.insight.200105 (PMC13135388; doi:10.1172/jci.insight.200105)
Supplement: Supplemental data [file jciinsight-11-200105-s297.pdf]

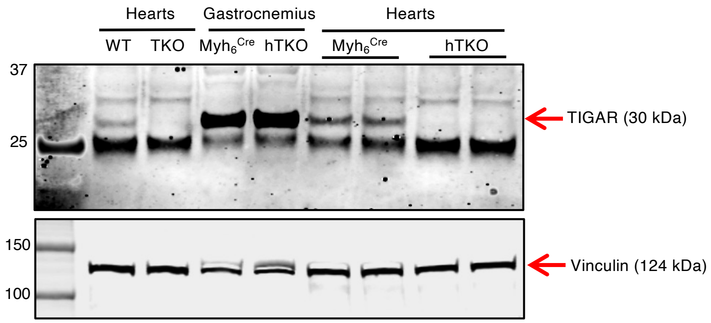

Supplemental Figure 1. Confirmation of TIGAR knockout in heart tissues. Western blot analysis demonstrating absence of TIGAR protein (30 kDa) in TKO and hTKO hearts compared with wild-type (WT) and Myh6^{Cre} control hearts. TIGAR protein expression was comparable in gastrocnemius muscle between Myh6^{Cre} and hTKO mice, confirming cardiac-specific nature of TIGAR deletion in the hTKO model. Vinculin (124 kDa) served as loading control. TKO and hTKO samples served as negative controls for TIGAR antibody specificity.

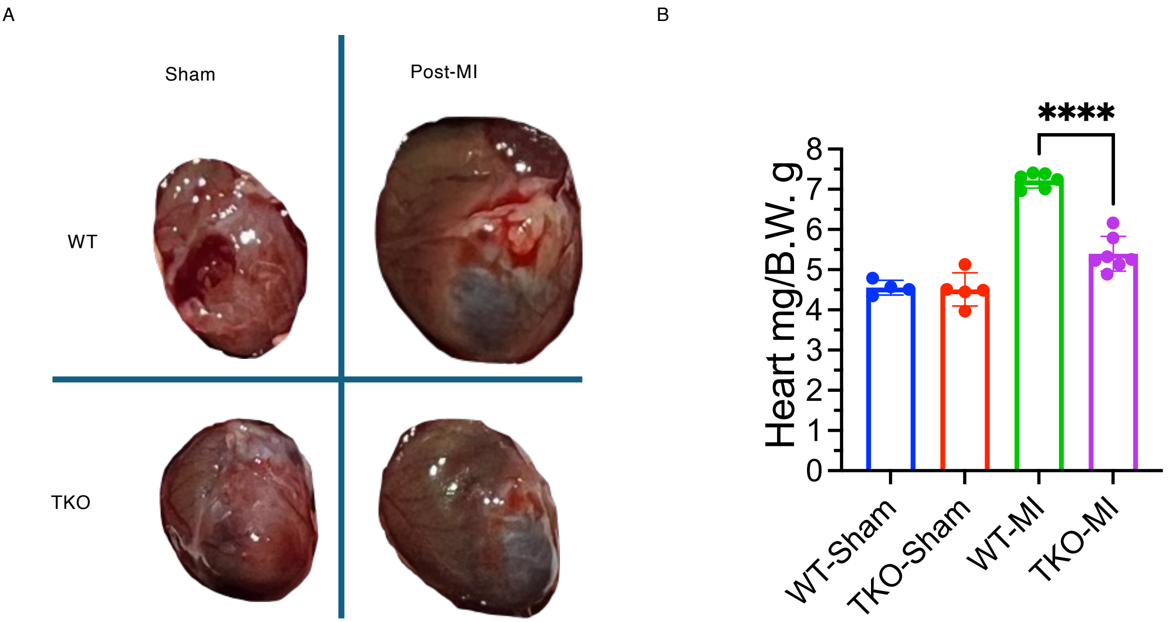

Supplemental Figure 2. TKO mice demonstrate reduced cardiac hypertrophy following myocardial infarction. A, Representative heart images from WT and TKO mice under sham operation and post-myocardial infarction (post-MI) conditions. **B,** Heart weight-to-body weight ratios (mg/g) in experimental groups. WT mice exhibited significant cardiac hypertrophy post-MI, which was attenuated in TKO mice. Data are presented as mean ± SD (n=6-7 per group). Statistical significance was determined by one-way ANOVA. ****P<0.0001.

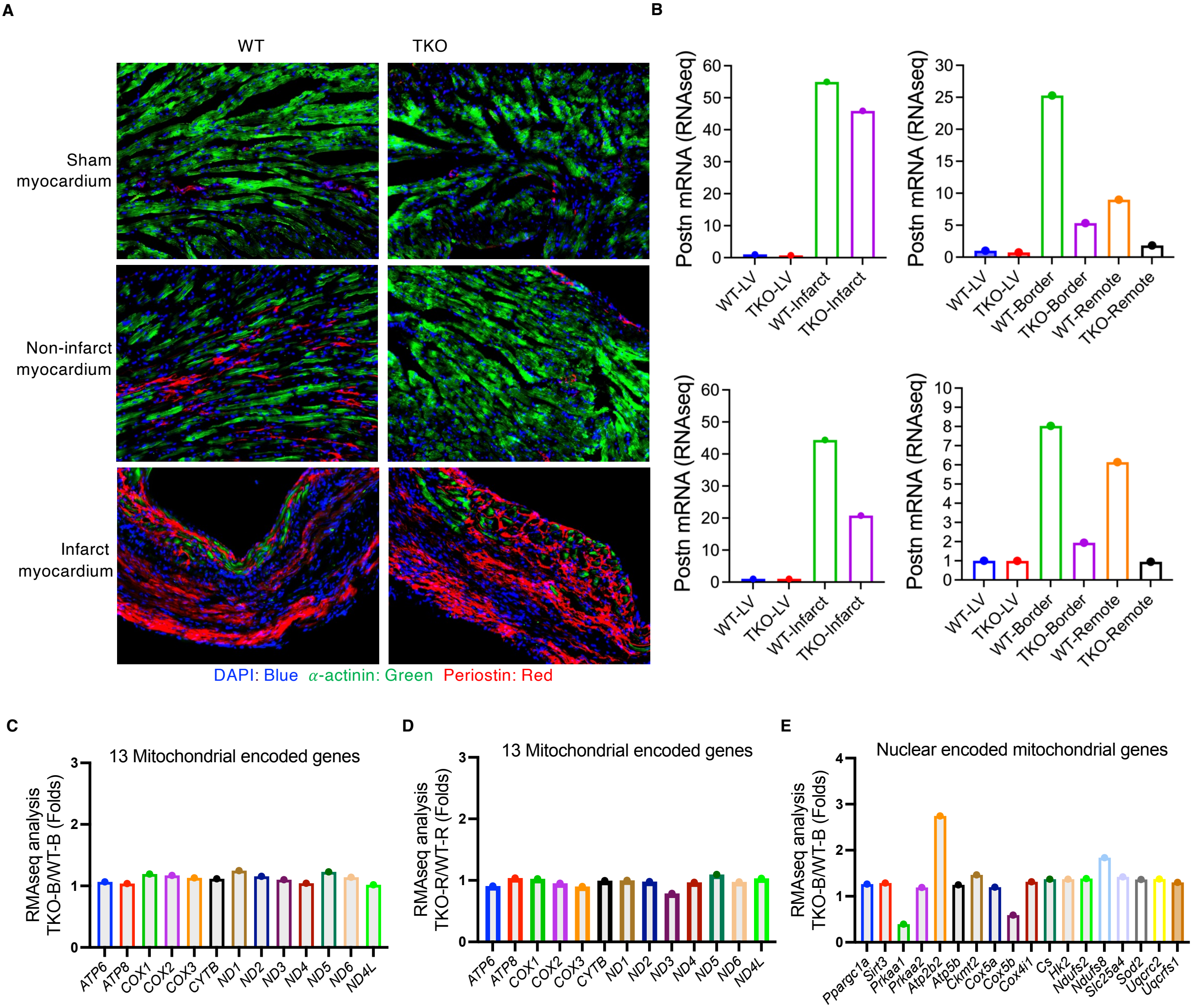

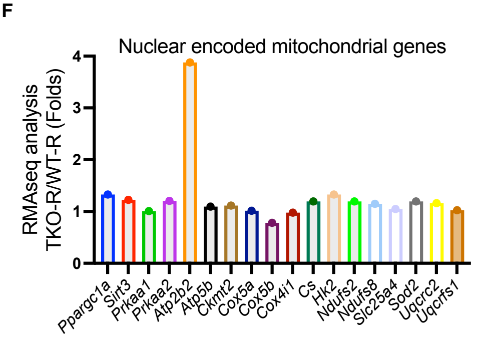

Supplemental Figure 3. Restricted fibrosis development in TKO mice 4 weeks post-MI. A, Representative immunofluorescence images of heart sections from WT and TKO mice showing sham myocardium, non-infarct myocardium, and infarct myocardium. Sections were stained for DAPI (blue, nuclei), α-actinin (green, cardiomyocytes), and periostin (red, fibrosis marker). **B,** RNA sequencing analysis of fibrosis marker genes *Postn* (periostin) and *Lgals3* (galectin-3) in left ventricle (LV), infarct zone, border zone, and remote zone of WT and TKO hearts. TKO hearts demonstrated reduced expression of fibrosis markers in non-infarct (remote) myocardium compared with WT hearts. **C,D,** RNA-seq analysis of mitochondrial-encoded genes from border zone (C) or remote zone (D). **E,F,** RNA-seq analysis of nuclear-encoded mitochondrial genes from border zone (E) or remote zone (F).

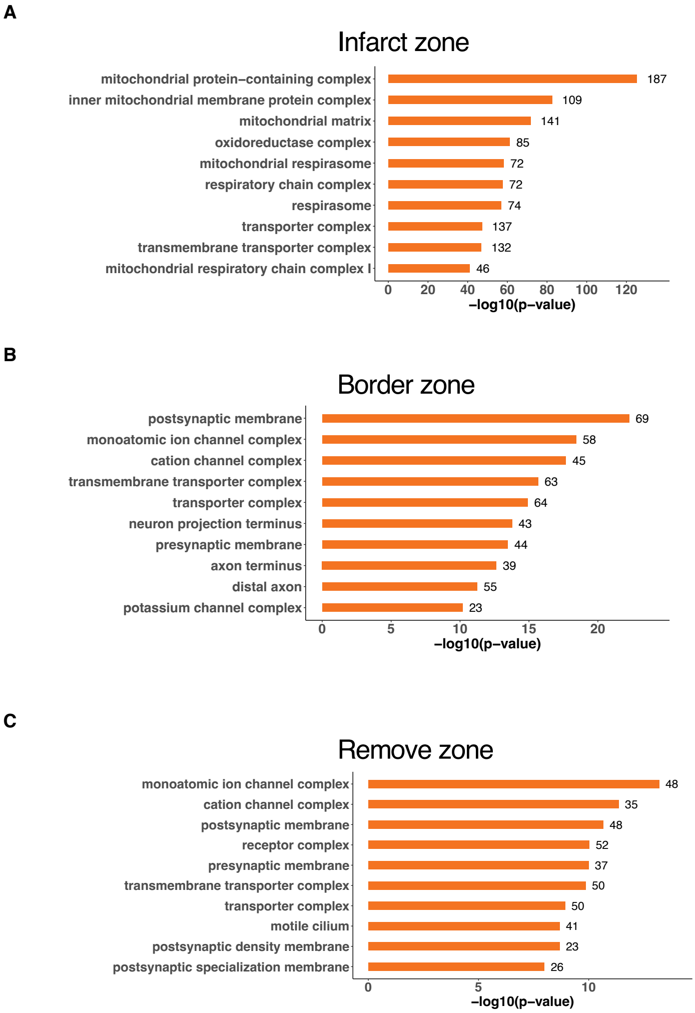

Supplemental Figure 4. Distinct pathway enrichment across infarct, border, and remote zones in WT and TKO hearts. Gene ontology pathway enrichment analysis of differentially expressed genes comparing WT and TKO mice in the infarct zone (A), border zone (B), and remote zone (C).

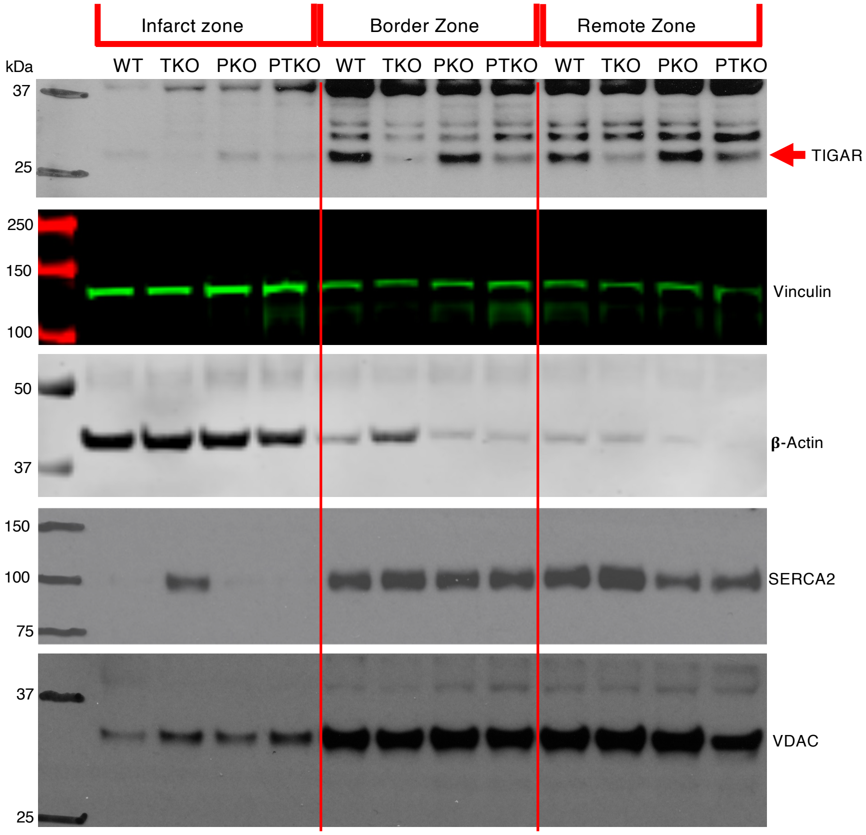

Supplemental Figure 5. SERCA2 protein preservation in TKO heart infarct zone post-MI. Western blot analysis of protein expression in infarct zone, border zone, and remote zone from WT, TKO, Parkin knockout (PKO), and Parkin/TIGAR double knockout (PTKO) hearts post-MI. SERCA2 (100 kDa) protein was preserved in infarct zone of TKO hearts but depleted in WT, PKO, and PTKO hearts. Tissue preparation: Hearts were homogenized in RIPA lysis buffer (sc-24948A) containing protease/phosphatase inhibitors (Halt™), 20 μM MG-132, and 20 μM ALLN; 30 μg total protein loaded per lane. TKO samples served as negative controls for TIGAR antibody specificity.

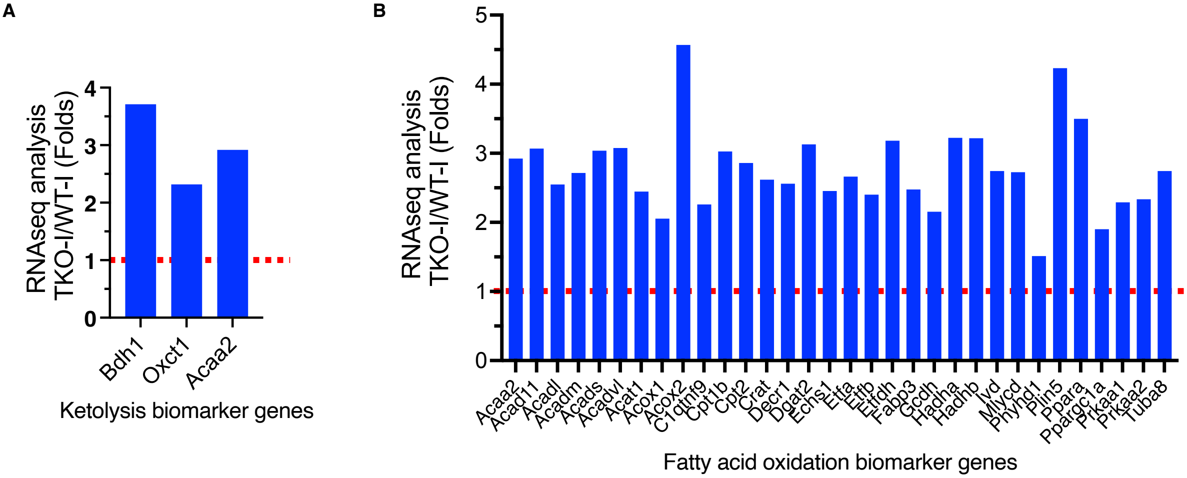

Supplemental Figure 6. Metabolic gene expression profiles in infarct zones. RNA sequencing analysis comparing gene expression ratios (TKO-Infarct/WT-Infarct) in infarct zones. **A,** Ketolysis biomarker genes (*Bdh1*, *Oxct1*, *Acaa2*). **B,** Fatty acid oxidation biomarker genes. Results demonstrate relative preservation of metabolic gene expression in TKO infarct zones compared with WT infarct zones. While these metabolic genes were downregulated in infarct zones of both genotypes compared with non-infarct tissue, fold changes >1 indicate that TKO hearts maintained relatively higher expression levels, suggesting preserved metabolic capacity following myocardial infarction.

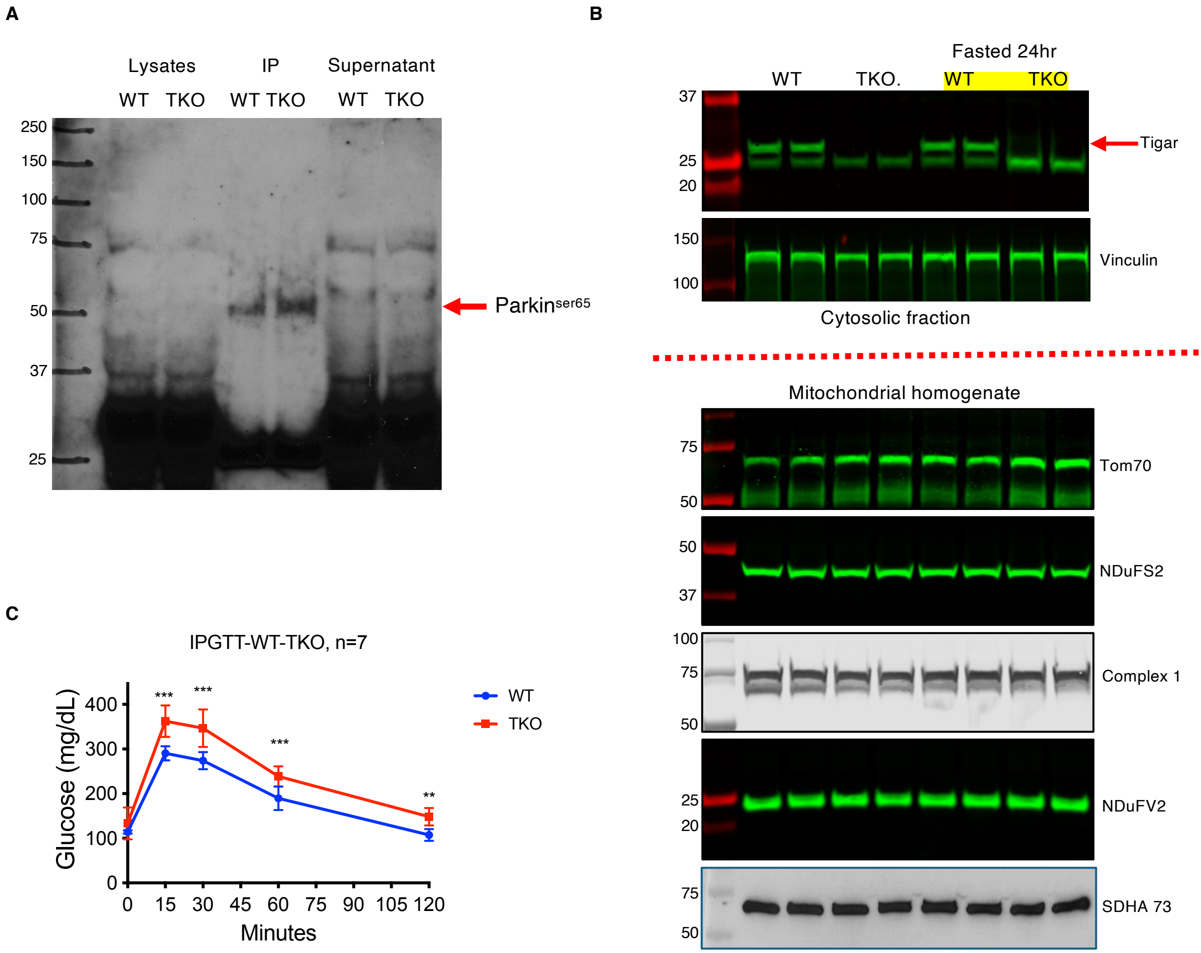

Supplemental Figure 7. Parkin phosphorylation and mitochondrial protein analysis in TKO hearts. A, Analysis of Parkin serine 65 phosphorylation in WT and TKO hearts. Heart tissues were homogenized in RIPA lysis buffer (composition as described in Supplementary Figure 4), and 2 mg lysate was subjected to immunoprecipitation (IP). Lysate lanes show 40 µg total protein (2% input) before immunoprecipitation. IP lanes represent samples immunoprecipitated with Parkin antibody and immunoblotted with phospho-Parkin Ser65 antibody. Supernatant lanes show post-IP samples (40 µg). **B,** Western blot analysis of mitochondrial proteins in cytosolic fractions and mitochondrial homogenates from fed and 24-hour fasted WT and TKO mice. Proteins analyzed include TIGAR (30 kDa), Vinculin (124 kDa), Tom70 (70 kDa), NDUFS2 (43 kDa), Complex I (75 kDa), NDUFV2 (24 kDa), and SDHA (73 kDa). Comparable levels of mitochondrial proteins were observed between WT and TKO hearts under normal conditions. **C,** Impaired glucose tolerance test in WT and TKO mice. Blood glucose levels over time in WT and TKO mice under normal chow conditions at 3 month of age. Data are presented as mean±SD; n=7 mice per group. Statictical significance was determined by Student's *t*-test. **P<0.01; ***P<0.0005.
