# Supplementary material for: TIGAR deficiency enhances cardiac resilience through epigenetic programming of Parkin expression
Source: JCI Insight. 2026 Feb 26;11(8):e200105. doi: 10.1172/jci.insight.200105 (PMC13135388; doi:10.1172/jci.insight.200105)
Supplement: Unedited blot and gel images [file jciinsight-11-200105-s298.pdf]

Full unedited gel for figure 1I

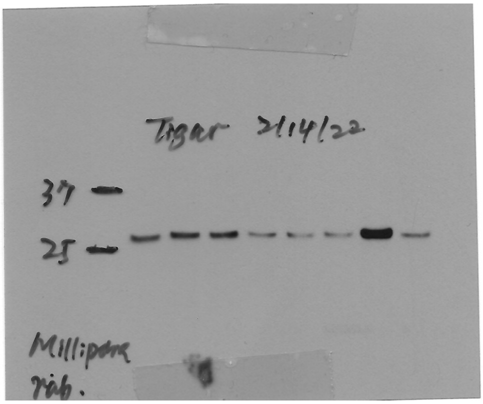

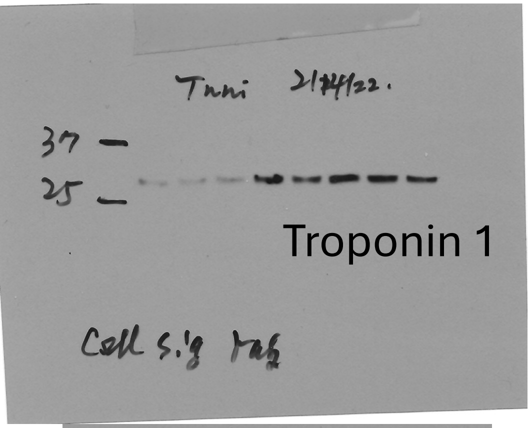

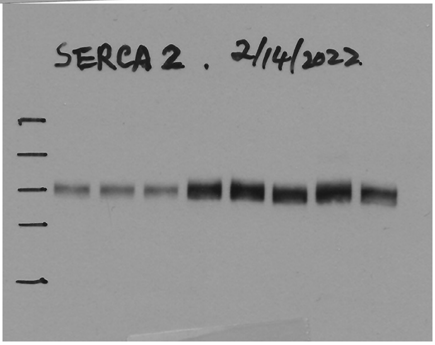

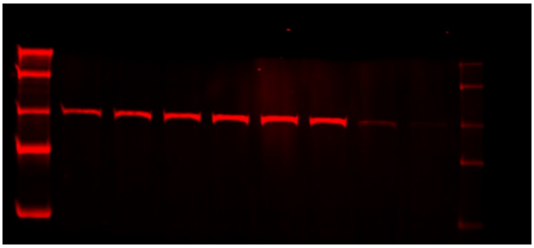

α-actinin1

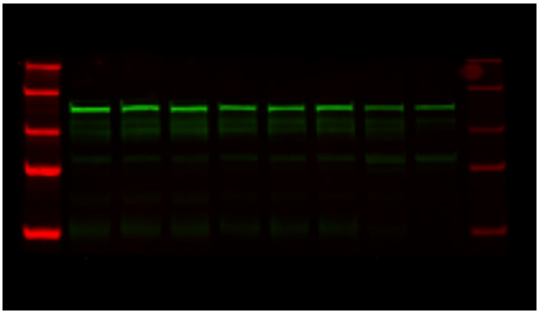

Vinculin

Full unedited gel for figure 2D

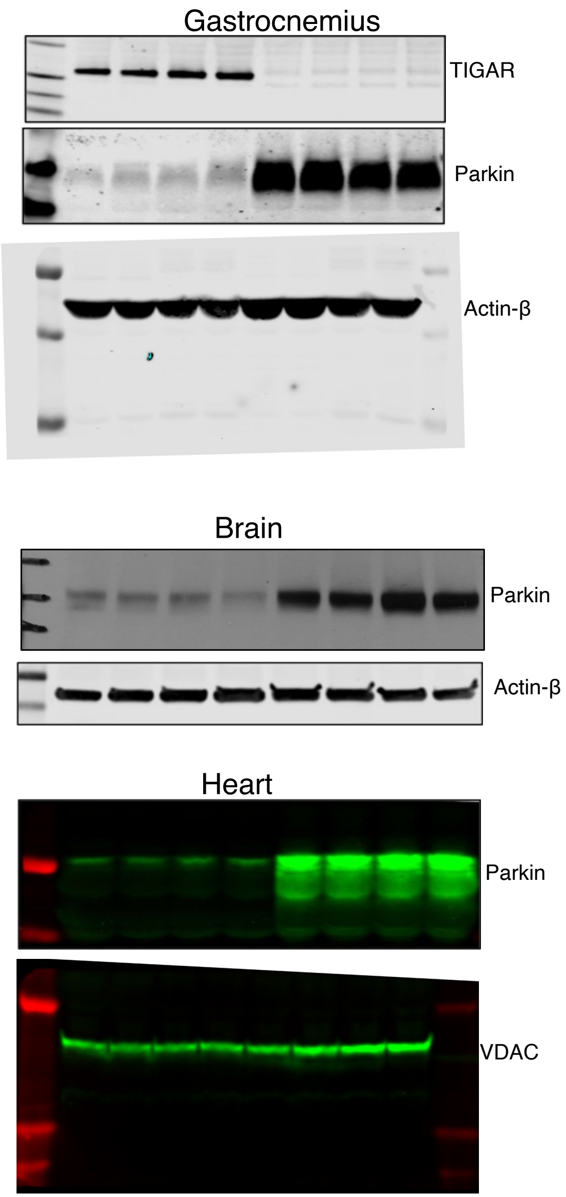

Full unedited gel for figure 2E

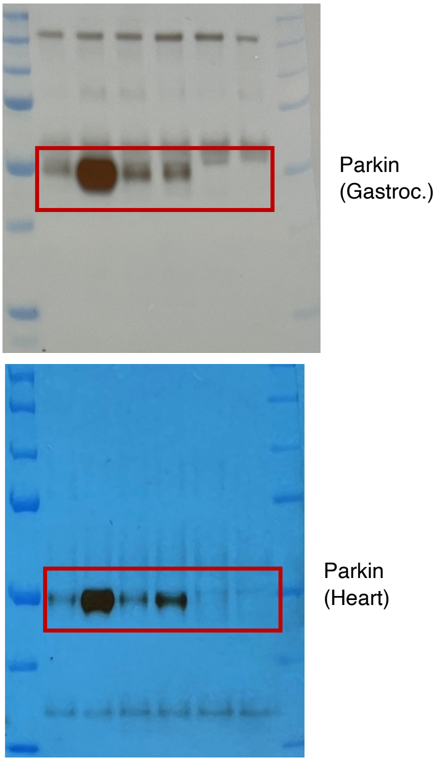

Full unedited gel for figure 2F

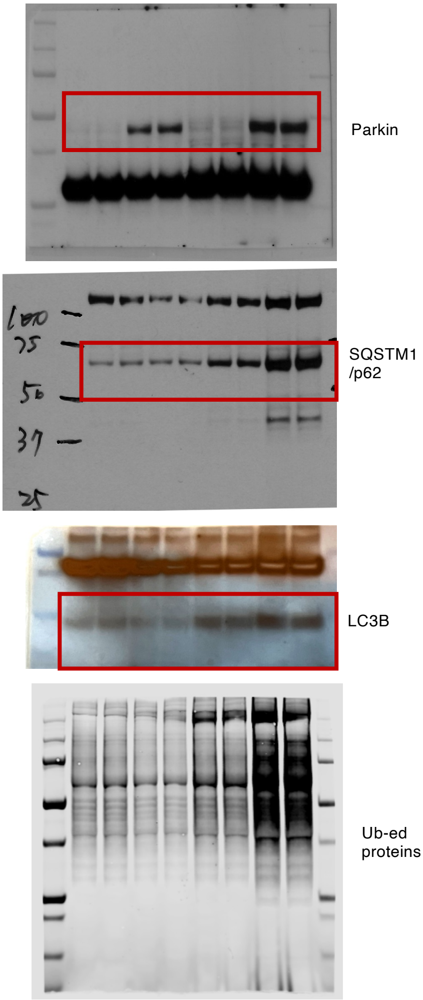

Full unedited gel for figure 2G

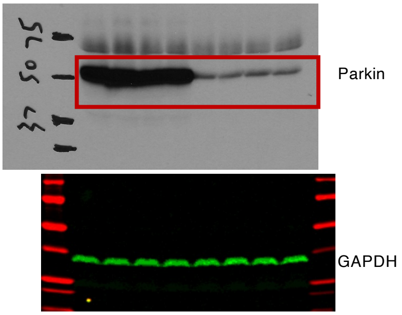

Full unedited gel for figure 2H

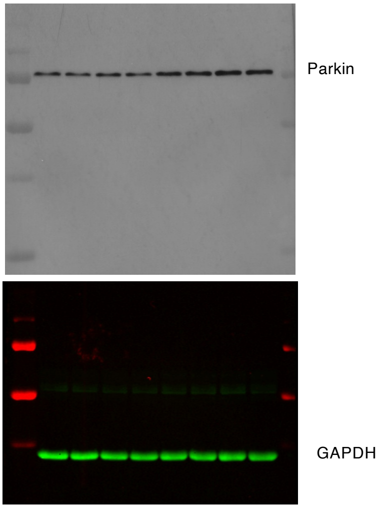

Full unedited gel for figure 3H

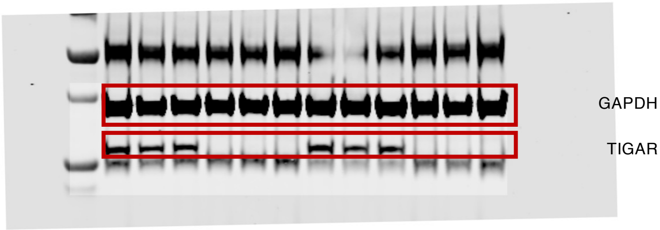

GAPDH

TIGAR

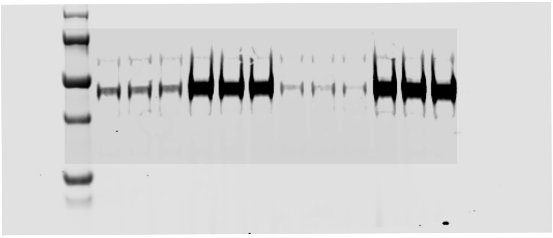

Parkin

Full unedited gel for figure 3I

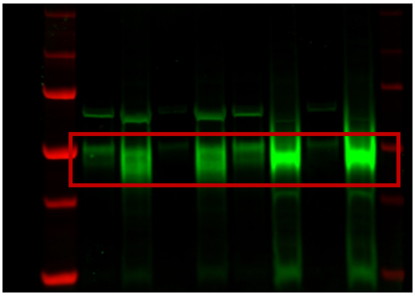

Parkin

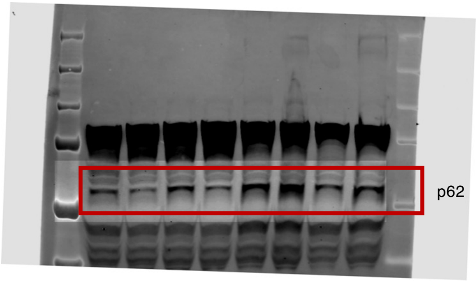

p62

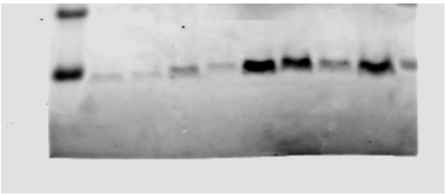

LC3B

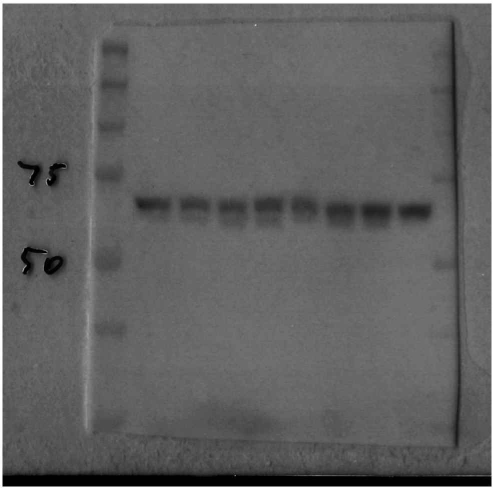

SDHA S

Full unedited gel for figure 4E

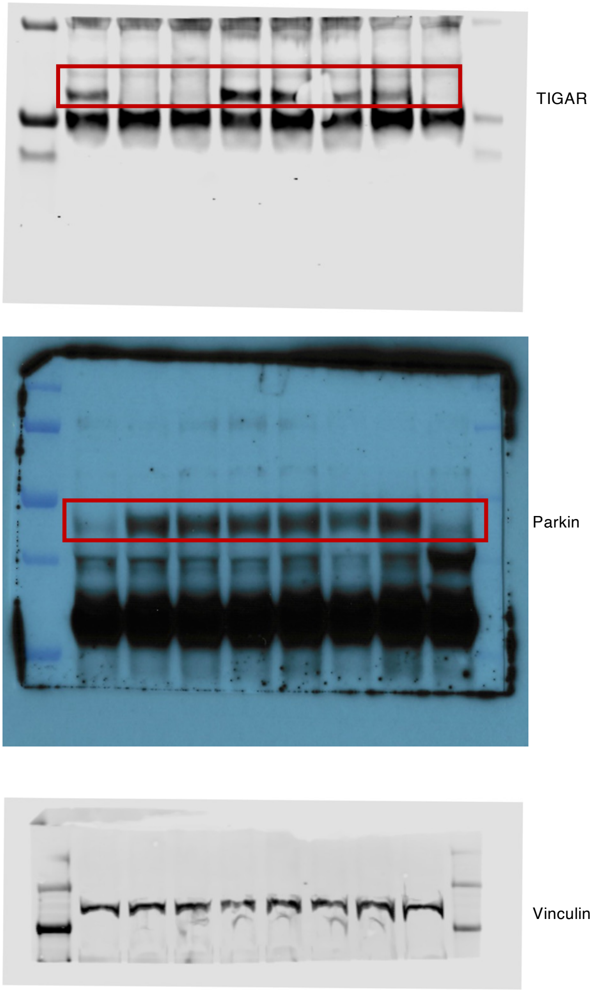

Full unedited gel for figure 5D

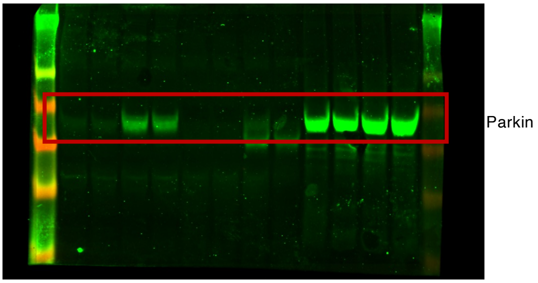

Full unedited gel for figure 5G

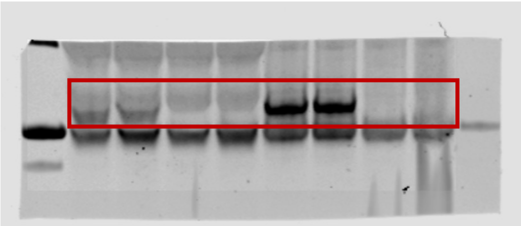

TIGAR

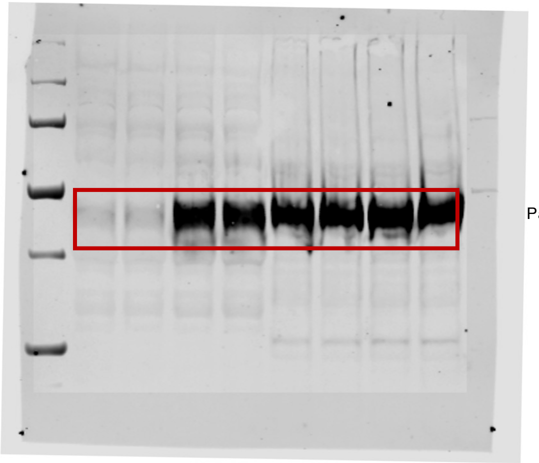

Parkin

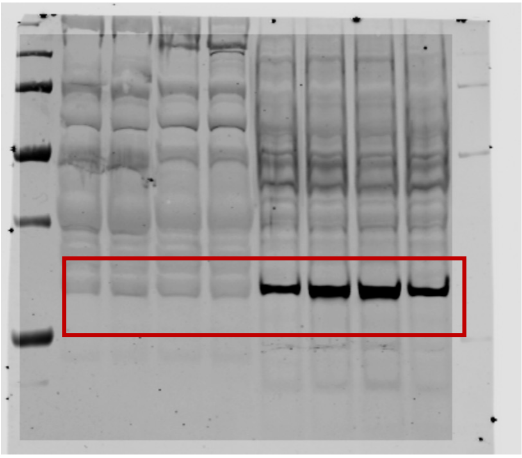

Pacrg

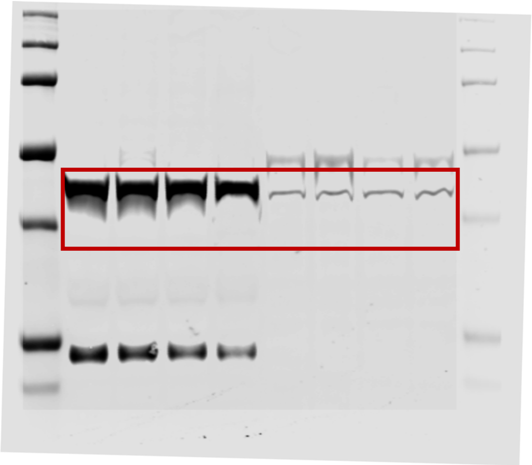

Ndufs2

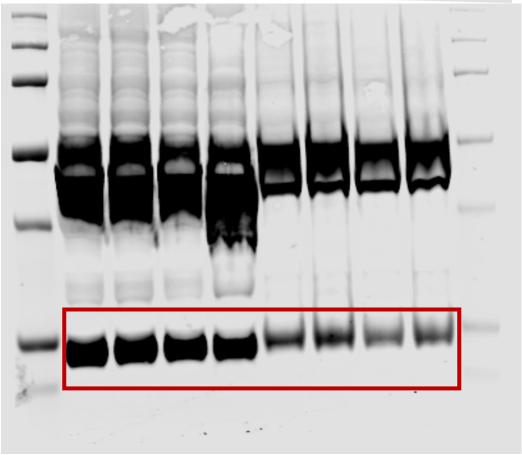

Ndufv2

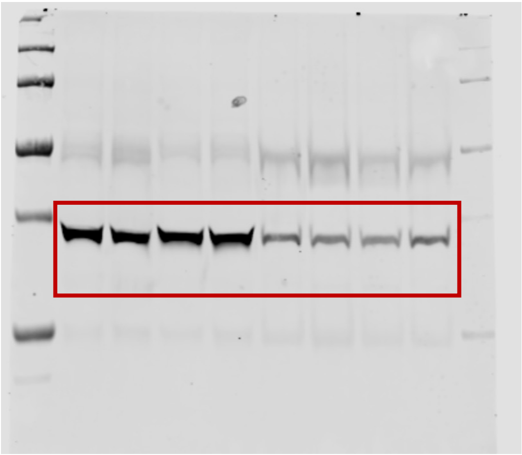

GAPDH

Full unedited gel for figure 6E

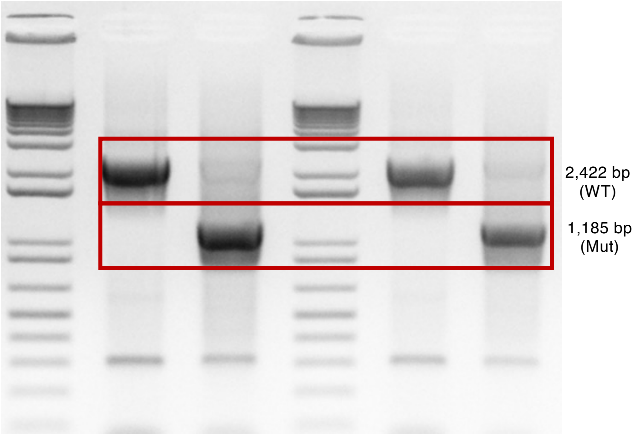

Full unedited gel for Supplemental figure 1

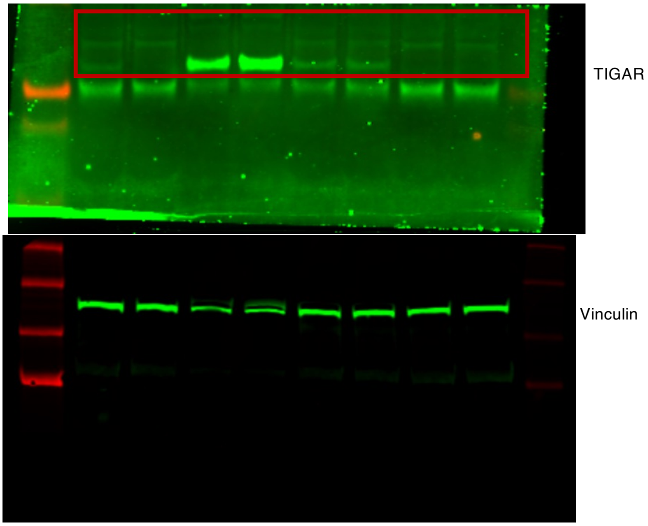

Full unedited gel for Supplemental figure 5

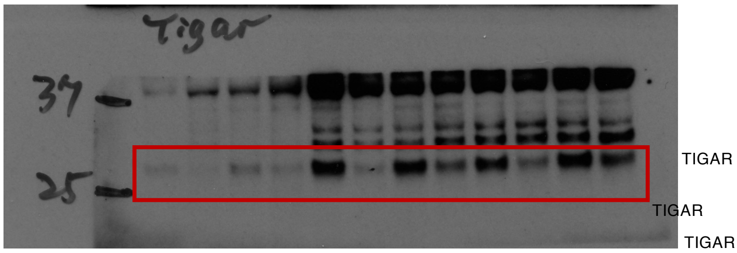

TIGAR

TIGAR

TIGAR

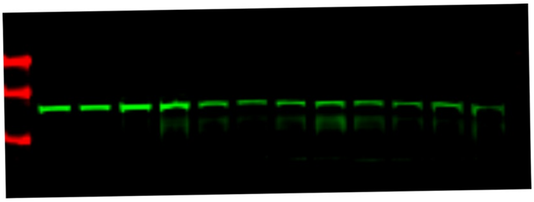

Vinculin

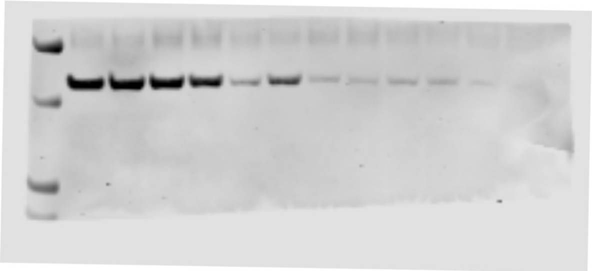

β-Actin

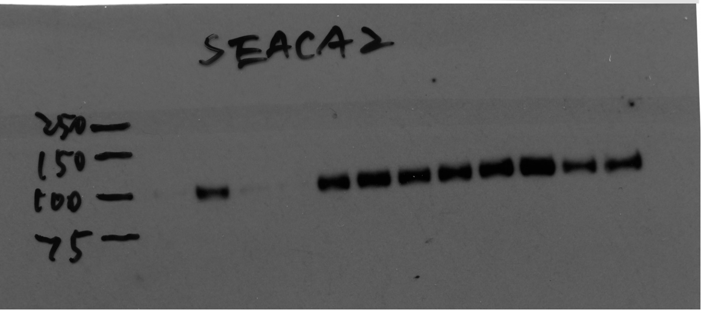

SERCA2

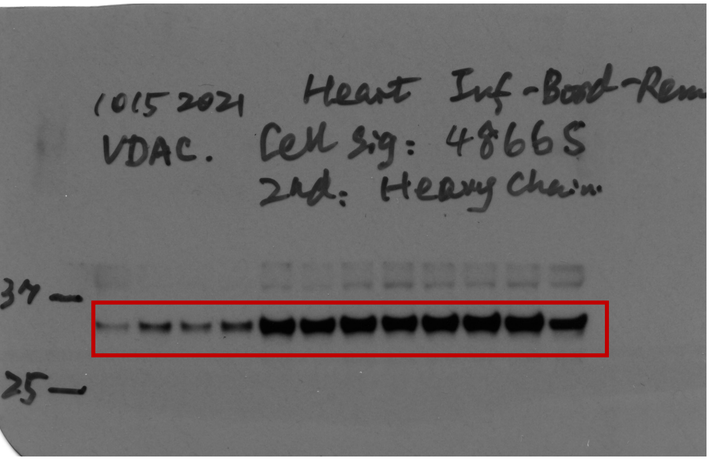

VDAC

Full unedited gel for Supplemental figure 7A

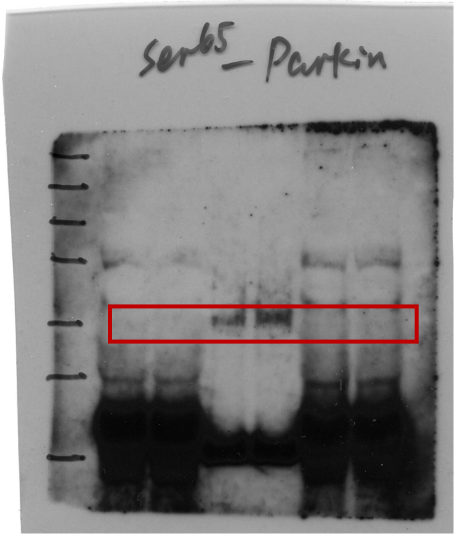

Parkin^{ser65}

Full unedited gel for Supplemental figure 7B

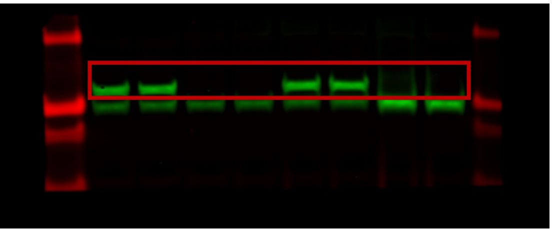

Tigar

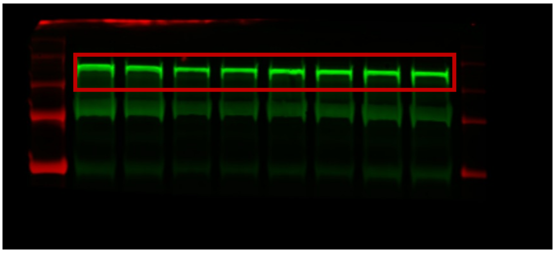

Vinculin

Full unedited gel for Supplemental figure 7B

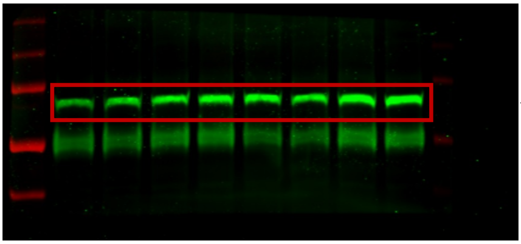

Tom70

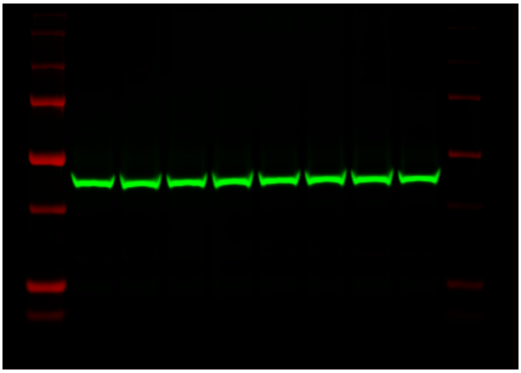

NDuFS2

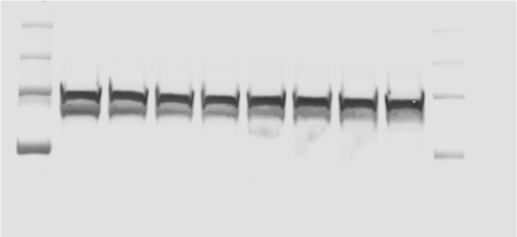

Complex 1

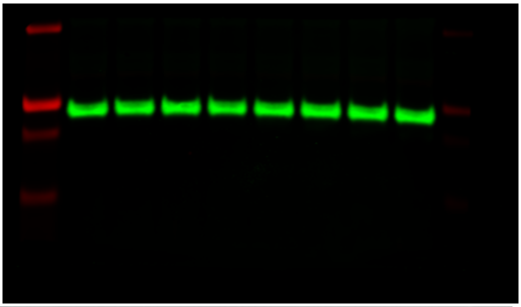

NDuFV2

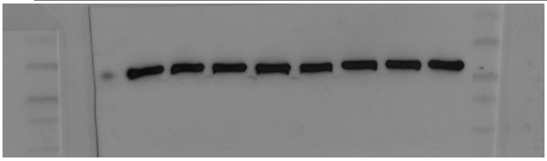

SDHA 73
